# Meissner Effect and Nonreciprocal Charge Transport in Non‐Topological 1T‐CrTe_2_/FeTe Heterostructures

**DOI:** 10.1002/adma.202520598

**Published:** 2026-02-05

**Authors:** Zi‐Jie Yan, Ying‐Ting Chan, Wei Yuan, Annie G. Wang, Hemian Yi, Zihao Wang, Lingjie Zhou, Hongtao Rong, Deyi Zhuo, Ke Wang, John Singleton, Laurel E. Winter, Weida Wu, Cui‐Zu Chang

**Affiliations:** ^1^ Department of Physics The Pennsylvania State University University Park Pennsylvania USA; ^2^ Department of Physics and Astronomy Rutgers University Piscataway New Jersey USA; ^3^ Materials Research Institute The Pennsylvania State University University Park Pennsylvania USA; ^4^ National High Magnetic Field Laboratory Los Alamos New Mexico USA

## Abstract

Interface‐induced superconductivity has recently been achieved by stacking a magnetic topological insulator layer on an antiferromagnetic FeTe layer. However, the mechanism driving this emergent superconductivity remains unclear. Here, we employ molecular beam epitaxy to grow a 1T‐CrTe_2_ layer, a 2D ferromagnet with a Curie temperature up to room temperature, on a FeTe layer. These 1T‐CrTe_2_/FeTe heterostructures show superconductivity with a critical temperature of ∼12 K. Through magnetic force microscopy measurements, we observe the Meissner effect on the surface of the 1T‐CrTe_2_ layer. Our electrical transport measurements reveal that the 1T‐CrTe_2_/FeTe heterostructures exhibit nonreciprocal charge transport behavior, characterized by a large magneto‐chiral anisotropy coefficient. The enhanced nonreciprocal charge transport in 1T‐CrTe_2_/FeTe heterostructures provides a promising platform for exploring the magnetically controllable superconducting diode effect.

## Graphical abstract

This work demonstrates interface‐induced superconductivity (*T*_c_ ∼12 K) with a robust Meissner effect and pronounced nonreciprocal transport in epitaxial 1T‐CrTe_2_/FeTe heterostructures, where 1T‐CrTe_2_ is a 2D ferromagnet, and FeTe is an antiferromagnetic metal. This finding establishes that topological surface states are not required to induce superconductivity in FeTe‐based heterostructures. The 1T‐CrTe_2_/FeTe heterostructures provide a platform for exploring magnetically controllable superconducting diode effects.

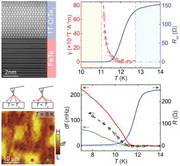

## 1 Main Text

Over the past few decades, emergent phenomena at the interface of two different materials have attracted significant research attention due to their novel and often unexpected physical properties. For example, a 2D electron gas and superconductivity have been observed at the LaAlO_3_/SrTiO_3_ interface [1, 2]; the enhanced superconductivity has been achieved in monolayer FeSe/SrTiO_3_ heterostructures [3, 4, 5, 6]. In addition, the interface can naturally induce the proximity effect, enabling one material to obtain the properties of its neighboring material [7, 8, 9]. This process can lead to the emergence of various exotic states of matter, including superconductivity, magnetism, and topologically nontrivial phases. Moreover, the formation of heterostructures inherently breaks the inversion symmetry at the interface, potentially giving rise to nonlinear responses [10, 11, 12, 13, 14, 15], such as nonreciprocal charge transport in superconducting heterostructures [16, 17, 18].

Recently, interface‐induced superconductivity and nonreciprocal charge transport have been observed in (Bi,Sb)_2_Te_3_/FeTe heterostructures [19, 20, 21, 22], where FeTe is an antiferromagnetic iron chalcogenide that is non‐superconducting without element doping [23, 24, 25] or tensile stress [26] and (Bi,Sb)_2_Te_3_ is a 3D topological insulator (TI) [27, 28]. Remarkably, this emergent superconductivity persists even after introducing magnetism into the TI layer and coexists with the ferromagnetism or antiferromagnetism in the magnetic TI layer [29, 30]. These magnetic TI/FeTe heterostructures provide a promising platform for exploring chiral Majorana physics and developing topological quantum computations. To date, interface‐induced superconductivity in FeTe‐based heterostructures has been observed exclusively in topological material/FeTe heterostructures [19, 20, 21, 22, 29, 30], with the mechanism behind this superconductivity remains unclear [19, 20, 21, 31, 32, 33, 34]. The large nonreciprocal charge transport in Bi_2_Te_3_/FeTe heterostructures has been attributed to the interplay between the induced superconductivity and the topological Dirac surface states of Bi_2_Te_3_ (Ref. [22]). These observations raise an important question: Is the topological order of the top layer essential for inducing superconductivity in FeTe‐based heterostructures? In other words, can superconductivity emerge in nontopological material/FeTe heterostructures, and if so, will the large nonreciprocal charge transport persist therein? To address these questions, it is necessary to find a nontopological material that can replace the TI layer while retaining the interface‐induced superconductivity.

**FIGURE 1:**
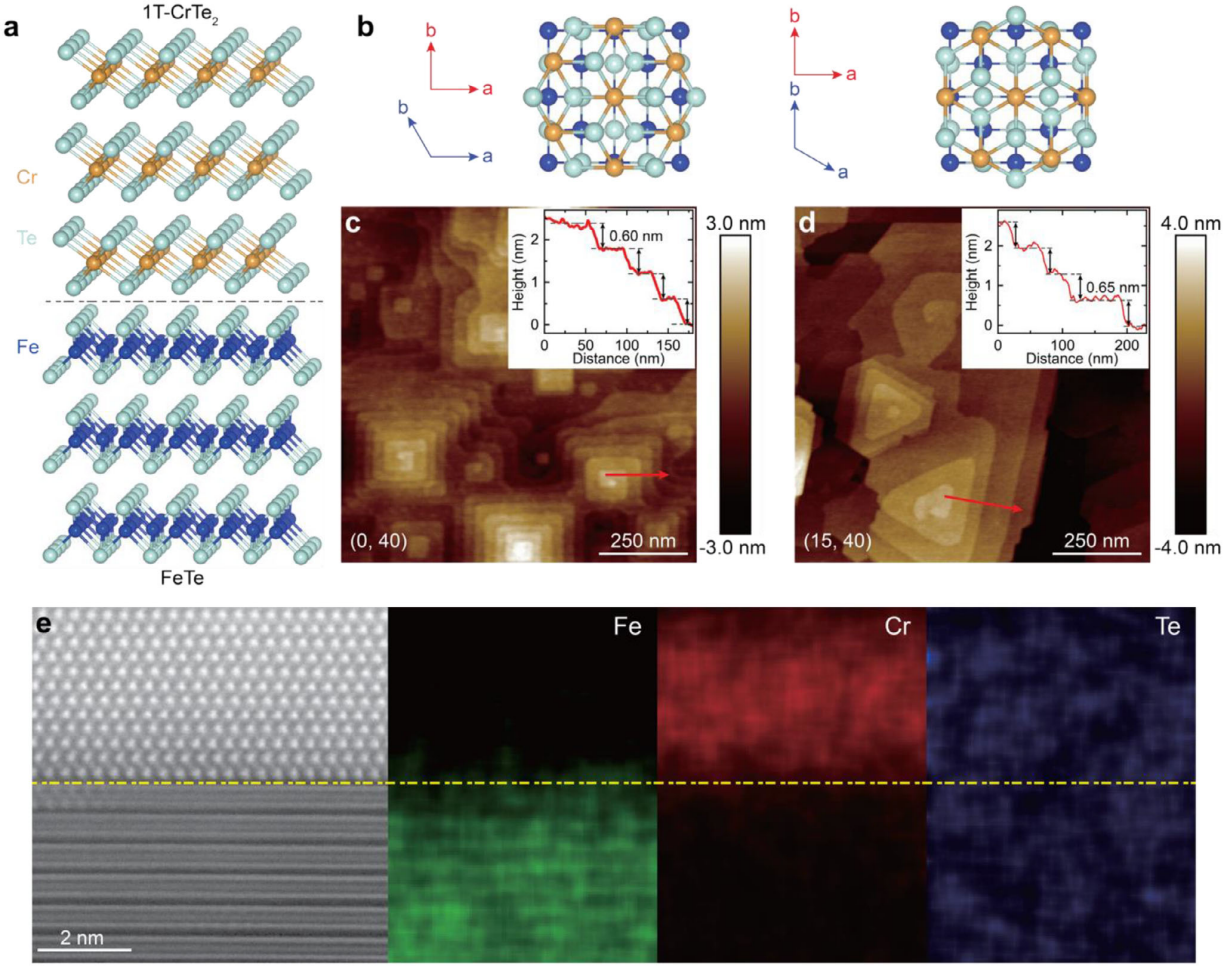
MBE‐grown 1T‐CrTe_2_/FeTe heterostructures on SrTiO_3_(100). (a) Schematic lattice structure of the 1T‐CrTe_2_/FeTe heterostructure. (b) Two possible stacking orientations of 1T‐CrTe_2_ on FeTe. The blue (red) arrows represent the crystal axes of the top 1T‐CrTe_2_ (bottom FeTe) layer. (c,d) 1 µm × 1 µm AFM images of 40 UC FeTe/SrTiO_3_(100) (c) and 15 TL CrTe_2_/40 UC FeTe/SrTiO_3_(100) (d). Insets: the height profiles along the red arrows. (e) Cross‐sectional STEM image of the 15 TL CrTe_2_/40 UC FeTe heterostructure with EDS mappings of Fe, Cr, and Te elements, respectively. The yellow dashed line indicates the sharp interface between 1T‐CrTe_2_ and FeTe.

1T‐CrTe_2_ is a layered ferromagnet with a trigonal crystal structure, where a Cr layer is sandwiched between two Te layers (Figure 1a). The Cr atoms exhibit long‐range ferromagnetic order along the *c*‐axis, and bulk 1T‐CrTe_2_ has a Curie temperature (*T*_Curie_) above room temperature [35]. Prior studies have shown that intrinsic ferromagnetism persists even when the thickness of 1T‐CrTe_2_ is reduced to a few atomic layers, achieved through either mechanical exfoliation [36] or epitaxial growth [37, 38]. Its trivial band structure, ferromagnetic property, and lattice structure similar to the Bi_2_Te_3_ family TI make 1T‐CrTe_2_ an ideal candidate to replace the magnetic TI layer in FeTe‐based heterostructures.

In this work, we employ molecular beam epitaxy (MBE) to grow a series of heterostructures by stacking *m* trilayer (TL) 1T‐CrTe_2_ on *n* unit‐cell (UC) FeTe (Figure 1a,b), referred to as the (*m*, *n*) heterostructures. Through in situ reflection high‐energy electron diffraction (RHEED) and ex situ atomic force microscopy (AFM) measurements, we demonstrate the epitaxy growth of 1T‐CrTe_2_ on FeTe despite their distinct in‐plane lattice rotational symmetries. Superconductivity emerges for *m* ≥ 1 and *n* ≥ 4, with the superconducting temperature (*T*_c_) increasing and saturating at ∼12 K as both *m* and *n* increase. Our magnetic force microscopy (MFM) measurements reveal the Meissner effect in 1T‐CrTe_2_/FeTe heterostructures, confirming the emergence of superconductivity. Further electrical transport measurements show enhanced nonreciprocal charge transport behavior, with the magneto‐chiral anisotropy coefficient much larger than previously reported values [22].

First, we characterize the evolution of surface morphology in the FeTe layer before and after the growth of the 1T‐CrTe_2_ layer. The AFM topography image of the 40 UC pristine FeTe layer on a heat‐treated SrTiO_3_(100) substrate reveals square‐shaped terraces (Figure 1c), corresponding to the tetragonal lattice structure of FeTe, with a measured step height of ∼0.60 nm between adjacent terraces (Figure 1c inset). This value is close to the bulk FeTe value of ∼0.65 nm (Refs. [39, 40]). After depositing 15 TL 1T‐CrTe_2_ layer on 40 UC FeTe, the topography transforms to triangular‐shaped terraces, consistent with the trigonal lattice structure of 1T‐CrTe_2_ (Figure 1d). The step height of the 1T‐CrTe_2_ terraces is ∼0.65 nm (Figure 1d inset), close to its bulk value of ∼0.62 nm (Refs. [40, 41]). We note that both the 1T‐CrTe_2_ and FeTe layers show sharp and streaky RHEED patterns during the MBE growth (Figure S1), confirming their highly ordered crystal structures.

Next, we conduct RHEED and AFM measurements on a series of (*m*, 40) heterostructures with varying *m*. As *m* increases, the RHEED patterns from the bottom 40 UC FeTe layer gradually diminish, while the RHEED patterns from the top 1T‐CrTe_2_ become more pronounced, eventually entirely replacing those of FeTe (Figure S1). We observe two sets of RHEED patterns for the 1T‐CrTe_2_ layer (Figure S1f), corresponding to diffractions along [1$\bar{1}$00] and [11$\bar{2}$0] directions, respectively. This observation indicates the twin‐boundary structure of the 1T‐CrTe_2_ layer, resulting from two possible epitaxial orientations (Figure 1b). This growth mode arises from the different in‐plane rotational symmetries of the 1T‐CrTe_2_ layer (i.e., sixfold) compared to the FeTe layer (i.e., fourfold). Besides RHEED, the AFM images show that the 1T‐CrTe_2_ layer first covers the edges of the square‐shaped FeTe terraces (Figure S2a–d), gradually extends across the entire films (Figure S2e), ultimately forms the triangular‐shapes terraces (Figure S2f). The sharp 1T‐CrTe_2_/FeTe interface is also confirmed by cross‐sectional scanning transmission electron microscopy (STEM) and corresponding energy dispersive X‐ray spectrometry (EDS) measurements (Figure 1e; Figure S3). The X‐ray diffraction (XRD) spectra and rocking curves of the (15, 40) heterostructure show sharp diffraction peaks from both 1T‐CrTe_2_ and FeTe layers, further validating the high quality of our 1T‐CrTe_2_/FeTe heterostructures (Figures S4 and S5).

**FIGURE 2:**
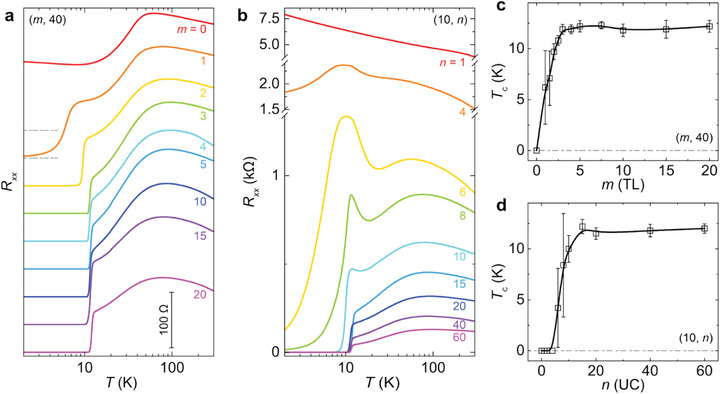
Interface‐induced superconductivity in 1T‐CrTe_2_/FeTe heterostructures. (a) *T* dependence of the sheet longitudinal resistance, *R*_xx_, of the (*m*, 40) heterostructures with 0 ≤ *m* ≤ 20. Each curve is shifted by 50 Ω. The two horizontal dashed lines indicate the zero resistance of the *m* = 0 and *m* = 1 samples. (b) *T* dependence of *R*_xx_ of the (10, *n*) heterostructures with 1 ≤ *n* ≤ 60. (c) *m* dependent superconducting temperature *T*_c_ of the (*m*, 40) heterostructures. (d) *n* dependent *T*_c_ of the (10, *n*) heterostructures. The *T*_c_ value is the temperature at which *R*_xx_ drops to 50% of its normal state resistance. The error bar of each sample is determined from the value difference between *T*_c,onset_ and *T*_c,0_.

Following sample characterization, we perform electrical transport measurements on two series of (*m*, *n*) heterostructures with varying *m* or *n*. Figure 2a shows the *R_xx_*‐*T* curves of the (*m*, 40) heterostructures. For *m* = 0, i.e., the pristine 40 UC FeTe layer, a hump feature is observed at *T* ∼ 60 K, corresponding to the paramagnetic‐to‐antiferromagnetic phase transition of the FeTe layer, known as its *Néel* temperature *T*_N_, consistent with prior studies [21, 29, 30]. For *m* = 1, a superconducting phase transition is observed, marked by a sudden drop of *R_xx_* near *T* = 7 K, which defines the superconducting onset temperature *T*_c,onset_. However, a zero‐resistance state is absent in the (1, 40) heterostructure down to *T* = 1.7 K, presumably due to the incomplete coverage of the top 1 TL CrTe_2_ layer, as confirmed by RHEED (Figure S1b) and AFM (Figure S2b) measurements. As *m* increases, the zero‐resistance state appears and persists at higher *T*, accompanied by a sharper superconducting phase transition in the *R_xx_*‐*T* curves. For *m* ≥ 3, the superconducting behavior becomes nearly uniform, with *T*_c_ saturating at ∼12 K (Figure 2a,c). Figure 2b shows the *R_xx_*‐*T* curves of the (10, *n*) heterostructures. For *n* < 4, the (10, *n*) heterostructures exhibit semiconducting behavior from room temperature down to *T* = 1.7 K, probably due to the inhomogeneity of the FeTe layer. For *n* = 4, *R_xx_* shows a sudden drop near *T* = 10 K, indicating a superconducting phase transition. This transition becomes sharper with increasing *n*, and the zero‐resistance state is observed for *n* ≥ 8. The *T*_c_ saturates at ∼12 K for *n* ≥ 15 (Figure 2b,d). The evolution of superconductivity with increasing *m* or *n* indicates that the induced superconducting state becomes spatially inhomogeneous when either the 1T‐CrTe_2_ or FeTe layer is thin, specifically *m* < 3 for *n* = 40 (Figure 2c) or *n* < 15 for *m* = 10 (Figure 2d).

**FIGURE 3:**
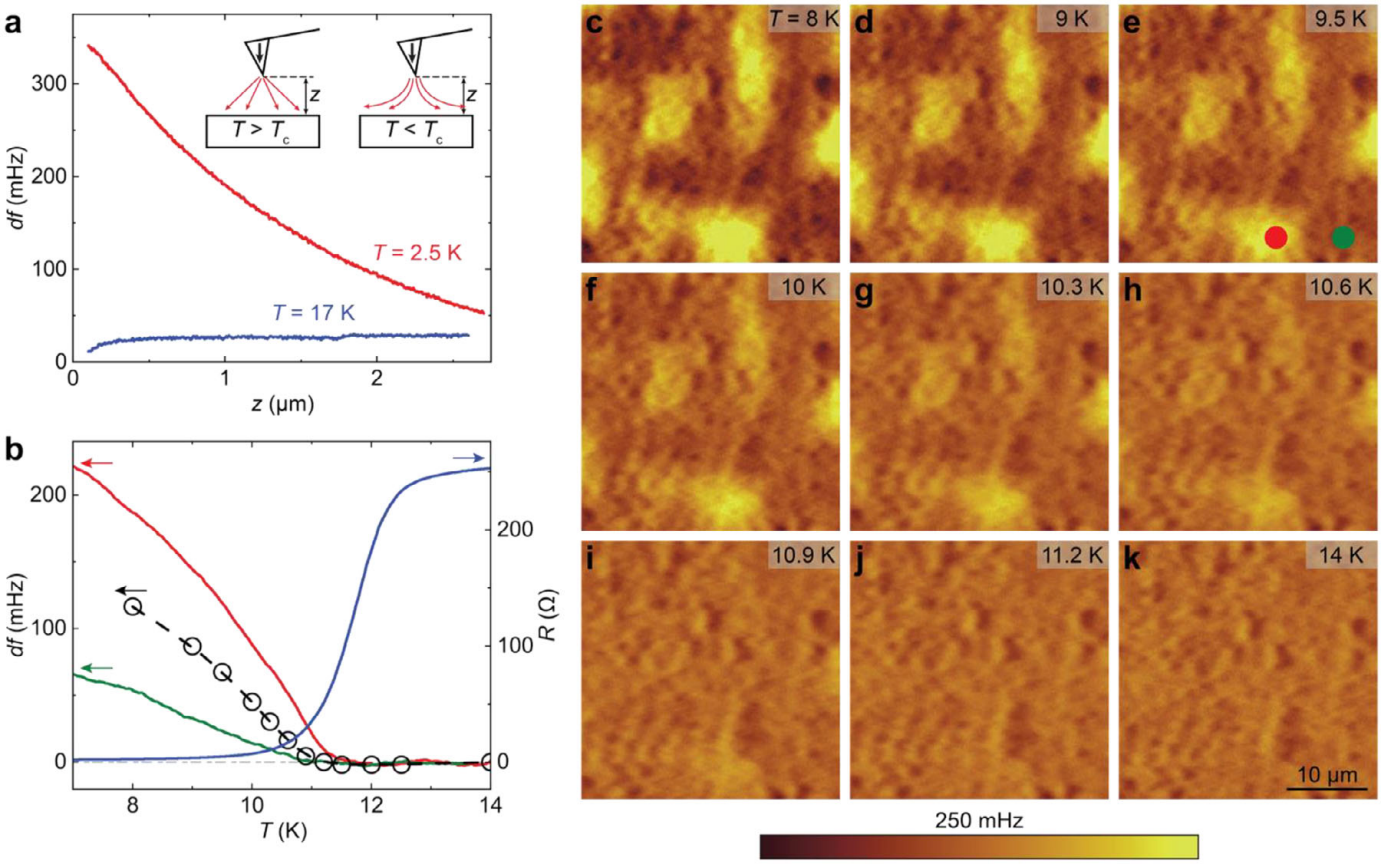
Meissner effect in 1T‐CrTe_2_/FeTe heterostructures. (a) *z*‐dependence of MFM frequency shift *df* measured on the (10, 20) heterostructure at *T* = 2.5 K (red) and *T* = 17 K (blue). Inset: Schematic of the Meissner effect experienced by the MFM tip. For *T* > *T*_c_, i.e., the normal state, the stray field of the magnetic tip can penetrate the heterostructure. However, for *T* < *T*_c_, i.e., the superconducting state, the Meissner effect partially expels the stray field and generates a repulsive force on the magnetic tip. (b) *T* dependence of *df* measured on the red and green dots in (e) (red and green curves), MFM contrasts (black circles), and in situ two‐terminal resistance *R* (blue curve) of the (20, 40) heterostructure. The MFM contrasts are defined as three times of the root mean square (RMS) value of each MFM image (Methods). (c‐k) MFM images of the (20, 40) heterostructure measured at *T* = 8 K (c), *T* = 9 K (d), *T* = 9.5 K (e), *T* = 10 K (f), *T* = 10.3 K (g), *T* = 10.6 K (h), *T* = 10.9 K (i), *T* = 11.2 K (j), and *T* = 14 K (k). During MFM measurements, the magnetic tip is ∼250 nm above the sample surface, and an external magnetic field of ∼0.2 T is applied.

To explore the spatial inhomogeneity of the superconducting state, we perform low‐temperature MFM experiments to probe the Meissner effect of the induced superconducting state. The Meissner effect is the spontaneous expulsion of the magnetic field in a superconductor, which is the other key characteristic of superconductivity besides the zero‐resistance state. The detection of Meissner's response would provide unambiguous evidence of the existence of superconductivity. Although the zero‐resistance state has been reported in numerous prior studies on FeTe‐based heterostructures, none reported the Meissner effect of the interface‐induced superconductivity [19, 20, 21, 29, 30, 31, 32, 33, 34]. MFM detection of the Meissner effect has been reported in various superconductors in either bulk crystals [42, 43, 44] or thin films [45, 46], where the magnetic tip experiences a repulsive force due to the expulsion of magnetic flux from the sample (Figure 3a inset). This repulsion results in a positive shift of the resonance frequency of the cantilever, *df* ∼ $\frac{\partial F(z)}{\partial z}$, which can be captured by the *df*‐*z* curve. Here *z* is the distance between the magnetic tip and the sample surface. Figure 3a shows a concave *df*‐*z* curve measured at a random point on the sample surface at *T* = 2.5 K, i.e., deep inside the superconducting state, clearly demonstrating the Meissner effect of our sample [42, 43, 44, 45, 46, 47]. For comparison, the *df* value remains nearly constant at *T* = 17 K (above superconducting *T*_c_), i.e., the normal state.

The spatial distribution of the magnetic response reflects the local phase rigidity in our heterostructures. To investigate the spatial variation of the Meissner repulsion, we take MFM images by scanning the MFM tip at ∼250 nm above the sample surface. Several brighter regions appear in the MFM image taken at *T* = 8 K with a 0.2 T magnetic field applied to stabilize the magnetic tip moment (Figure 3c). This result reveals a nonuniform Meissner's response over a length scale of ∼10 µm. We find that the magnetic contrast decreases gradually with increasing temperature and eventually disappears above *T*_c_ (∼11 K) (Figure 3c–k), which is determined by in situ two‐terminal resistance *R* (Figure 3b). Moreover, the spatial distribution of the Meissner response remains nearly unchanged after thermal cycling across superconducting *T*_c_ or under varying applied magnetic fields (Figures S6 and S7). This robustness indicates that the nonuniform distribution is not an extrinsic effect but instead likely associated with static features intrinsic to the 1T‐CrTe_2_/FeTe heterostructures, such as local thickness variations and structural defects.

To confirm that the MFM contrast comes from the inhomogeneity of the Meissner effect, we measure single‐point frequency shift *df* by positioning the MFM tip at locations with bright and dark contrast (Figure 3e) while cooling the sample through *T*_c_ (Figure 3b). The *df* signal remains constant above *T*_c_, then suddenly rises near *T* = 11 K, marking the onset of the Meissner effect below *T*_c_. The observation of the Meissner effect in both locations demonstrates the presence of superconductivity in all locations, and the observed MFM contrast is due to the inhomogeneous Meissner effect. Although spatial inhomogeneity is evident in the Meissner response, the entire film enters the superconducting state for *T* < *T*_c_. The onset of the Meissner repulsion at both the red and green dots occurs at the same temperature within our measurement resolution (Figure 3b), indicating that the global phase coherence is established across different regions of the 1T‐CrTe_2_/FeTe heterostructure. This finding is consistent with the sharp superconducting transition observed in our electrical transport measurements (Figure 2a). In other words, the weaker repulsion at the green dot likely reflects a smaller superconducting volume or lower superfluid density than the red dot (Figure 3b,e). Indeed, superconductivity in the regions with low superfluid density would be suppressed in the ultrathin limit, resulting in disconnected superconducting islands, i.e., finite resistance below the mean‐field transition temperature. Therefore, the observed inhomogeneity of superfluid density provides a natural mechanism for the thickness dependence of the zero‐resistance state. To further examine the spatial distribution of Meissner response in thinner samples, we perform MFM measurements on (1.5, 40) and (10, 10) heterostructures (Figures S8 and S9). Compared to the heterostructures with (*m*, *n*) = (20, 40) and (10, 20) (Figure 3), we find that reducing either *m* or *n* results in a much weaker Meissner response, a shorter inhomogeneity length scale, and a lower *T*_c_, indicating the formation of small superconducting patches with weak links. This scenario is consistent with the broader superconducting transition observed in thinner samples (Figure 2).

The zero‐resistance state observed in electrical transport and the Meissner effect detected through MFM unambiguously confirm the interface‐induced superconductivity in 1T‐CrTe_2_/FeTe heterostructures. This correlation between the Meissner effect and the zero‐resistance state highlights the robustness of the superconducting phase. Observing the Meissner effect in the 1T‐CrTe_2_/FeTe heterostructures provides valuable insights into the nature of the emergent superconductivity. First, despite spatial inhomogeneity, the strong Meissner response observed across the entire scanned area indicates that the emergent superconductivity is not confined to local regions or strictly limited to the CrTe_2_/FeTe interface but instead extends throughout the film (Figure 3). Second, the robustness of the Meissner response against thermal cycling and applied magnetic fields points to its origin in static features intrinsic to the CrTe_2_/FeTe heterostructures, including local strain variations, structural defects, or interface charge transfer (Figures S6 and S7). Moreover, as *T* decreases, the emerging Meissner effect indicates a strengthening of superconducting coherence, which is expected to enhance the nonreciprocal charge transport behaviors in 1T‐CrTe_2_/FeTe heterostructures.

**FIGURE 4:**
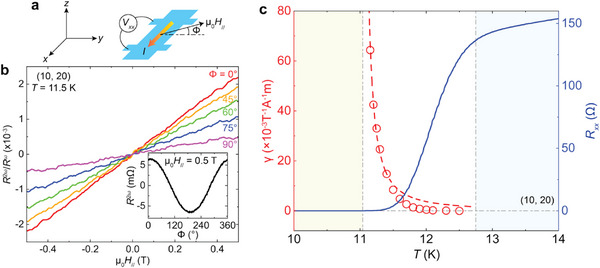
Nonreciprocal charge transport in the (10, 20) heterostructure. (a) Schematic of the nonreciprocal charge transport measurement setup. The angle *ϕ* is defined between the in‐plane magnetic field μ_0_ *H*_||_ and the *y*‐axis ($\hat{y}$) direction. (b) μ_0_ *H*_||_ dependence of second‐harmonic response (i.e., *R* ^2ω^/*R* ^ω^) measured under different *ϕ* and at *T* = 11.5 K. Inset: *ϕ* dependence of the second‐harmonic resistance *R* ^2ω^ measured at *T* = 11.5 K under a fixed μ_0_ *H*_||_ = 0.5 T. (c) *T* dependence of the magneto‐chiral anisotropy coefficient *γ* (red circles) and the sheet longitudinal resistance *R*_xx_ (blue curve). The red dashed curve is fitted by the formula *γ* = *β*(*T* − *T*_BKT_)^−1.5^, where *T*_BKT_ is the BKT superconducting transition temperature, and *β* is a fitting coefficient. The large nonreciprocal charge transport occurs only during the superconducting transition regime.

Next, we investigate the nonreciprocal charge transport in our 1T‐CrTe_2_/FeTe heterostructures. As noted above, the 1T‐CrTe_2_/FeTe interface inherently breaks the inversion symmetry. When an external in‐plane magnetic field μ_0_ *H*_||_ is applied to break the time‐reversal symmetry, a nonlinear term is expected to appear in the longitudinal voltage *V_xx_* (Refs. [14, 16]) (Figure 4a).

(1) $$V_{xx}=R^{\omega }\;I+\gamma R^{\omega }\left[\left(\mu_{0}\vec{H_{∥}}\times \hat{z}\right)\cdot \vec{I}\right]I$$

Here $\hat{z}$ is the normal direction of the sample plane, *R* ^ω^ is the first‐harmonic longitudinal resistance, and γ is the magneto‐chiral anisotropy coefficient [16]. The γ value quantifies the strength of nonlinear and nonreciprocal charge transport behaviors. When μ_0_ *H*_||_ is confined within the sample plane, i.e., perpendicular to $\hat{z}$, Equation (1) simplifies to *V_xx_* = *R* ^ω^ *I* + γ*R* ^ω^μ_0_ *H*_∥_cos *ϕ* *I* ^2^, where *ϕ* is the angle between μ_0_ *H*_||_ and $\hat{y}$ (Figure 4a). The second term in this expression accounts for the nonreciprocal charge transport, also known as the second‐harmonic signal, due to its quadratic dependence on the applied current *I*. The second harmonic resistance is thus given by $R^{2\omega }=\frac{\gamma R^{\omega }\mu_{0}H_{∥}\cos\phi I_{0}}{\sqrt{2}}\;$, where *I*_0_ is the effective value of the applied alternating current (a.c.) (Supporting Information).

To verify the presence of nonreciprocal charge transport in our 1T‐CrTe_2_/FeTe heterostructures and optimize the second harmonic measurement setup (Methods), we first measure *ϕ*‐dependent *R* ^2ω^ and observe a sinusoidal dependence (Figure 4b inset). For each fixed *ϕ*, *R* ^2ω^/*R* ^ω^ is consistently proportional to μ_0_ *H*_||_ (Figure 4b). This linear dependence is unaffected by the amplitude and frequency (Figure S10) of the applied a.c. current. These observations align with the above‐mentioned theoretical derivations, providing evidence for the emergence of the nonreciprocal charge transport in our 1T‐CrTe_2_/FeTe heterostructures. As *I*_0_ increases from 100 µA to 2 mA, the signal‐to‐noise ratio of the *R* ^2ω^‐μ_0_ *H*_||_ curves increases (Figure S10a) due to its linear dependence on *I*_0_. However, a larger excitation current inevitably suppresses the emergent superconductivity, as suggested by a monotonic increase in *R* ^ω^ (Figure S10b inset), leading to smaller γ values (Figure S10b). Therefore, we use the excitation current *I*_0_ of ∼500 µA with a frequency of ∼6.447 Hz in our following second‐harmonic transport measurements.

Next, we investigate the nonreciprocal charge transport at varying temperatures. For the (10, 20) heterostructure, its Berezinskii‐Kosterlitz‐Thouless (BKT) transition temperature *T*_BKT_ is ∼11.04 K, determined by fitting its *R_xx_*‐*T* curve with the Halperin–Nelson formula [48], $R_{xx}\propto \exp\left(-2b\sqrt{\frac{T_{c}-T}{T-T_{BKT}}}\right)$, where *b* is a fitting parameter. For *T* < *T*_BKT_, we find that *R* ^2ω^ vanishes along with *R* ^ω^, consistent with the zero‐resistance state (Figure S11a). For *T*_BKT_ < *T* < *T*_c,onset_, i.e., the superconducting transition regime where the superconducting order parameter appears, the nonreciprocal charge transport arises due to the fluctuating superconducting order parameter (Figure S11b–d). In contrast, for *T* > *T*_c,onset_, *R* ^2ω^ becomes negligible again (Figure S11e). The linear relationship between *R* ^2ω^ and μ_0_ *H*_||_ is only observed within the superconducting transition regime, i.e., *T*_BKT_ < *T* < *T*_c,onset_. In this regime, electrons begin to form Cooper pairs, shifting the energy scale of the electronic system to change from the Fermi energy (*E*_F_ ∼ eV) to the superconducting gap size (Δ ∼ meV). Therefore, the nonreciprocal charge transport, driven by the spin‐orbit interaction and the Zeeman effect, becomes relatively significant compared to the normal states for *T* > *T*_c,onset_ (Ref. [16]). With increasing *T*, γ gradually decreases and shows a slight deviation from the expected γ ∝ (*T* − *T*_BKT_)^−1.5^ dependence (Figure 4c). We attribute this deviation to a self‐heating‐induced thermal gradient, which increasingly affects *R* ^2ω^ as *R* ^ω^ becomes larger at elevated *T* (Figure S12).

The magneto‐chiral anisotropy coefficient γ of the (10, 20) heterostructure diverges as *T* approaches *T*_BKT_, reaching a maximum value of ∼64.3 × 10^−3^ T^−1^∙A^−1^m at *T* = 11.15 K (Figure 4c). This value is an order of magnitude larger than that of the Bi_2_Te_3_/FeTe heterostructures (Ref. [22]). For Bi_2_Te_3_/FeTe heterostructures, the origin of such large nonreciprocal transport is attributed to the Dirac surface states of the Bi_2_Te_3_ layer [22]. However, our second harmonic results suggest that the nonreciprocal transport persists and is even enhanced when the nontopological material 1T‐CrTe_2_ replaces the Bi_2_Te_3_ layer. This observation indicates that the large nonreciprocal charge transport in FeTe‐based heterostructures may arise from the FeTe layers near the interface, which become superconducting after the deposition of various top layers. We also perform second harmonic transport measurements on the (5, 40) and (3, 40) heterostructures (Figure S13). Similar behaviors and comparable thickness‐independent γ values confirm the interfacial origin of the emergent superconductivity in our 1T‐CrTe_2_/FeTe heterostructures.

Finally, we investigate the ferromagnetic properties of our superconducting 1T‐CrTe_2_/FeTe heterostructures. Figure S14a shows the Hall traces of the (10, 20) heterostructure at different temperatures. At *T* = 5 K, i.e., below its *T*_c_ (∼11.5 K), the Hall resistance *R_yx_* remains zero across the entire external magnetic field μ_0_ *H*_⊥_ range, consistent with the zero‐resistance state. For *T* > *T*_c_, a non‐zero *R_yx_* with a clear hysteresis loop appears during the μ_0_ *H*_⊥_ sweep and persists up to T ∼ 200 K, indicating ferromagnetism with *T*_Curie_ ∼ 200 K. Figure S14b summarizes its anomalous Hall resistance *R* ^AHE^ and coercive field μ_0_ *H*_c_ as a function of *T*. Similar results are observed in more heterostructures with different *m* and *n* (Figure S15). In addition, a sign reversal of *R* ^AHE^ and the topological Hall effect are observed in the pristine 15 TL 1T‐CrTe_2_ film (Figure S16), consistent with the prior studies [49, 50]. However, both behaviors are absent in other (*m*, *n*) heterostructures, presumably due to differences in interfacial strain between 1T‐CrTe_2_/FeTe and 1T‐CrTe_2_/SrTiO_3_(100) interfaces.

To summarize, we use MBE to grow 1T‐CrTe_2_/FeTe heterostructures and observe the emergent superconductivity in these heterostructures. Our MFM measurements reveal the Meissner effect, and our second‐harmonic measurements show nonreciprocal charge transport with a large γ value. The observations of the Meissner effect and nonreciprocal charge transport in our 1T‐CrTe_2_/FeTe heterostructures confirm that the topological surface states of the top layer are not a prerequisite for creating superconductivity in FeTe‐based heterostructures. Our results indicate that the FeTe layer near the interface is most likely responsible for the emergent superconductivity. We hypothesize that the Te element may be crucial for the formation of the superconductivity due to its consistent presence in the top layer of various FeTe‐based superconducting heterostructures [19, 20, 21, 22, 29, 30, 31, 32, 33, 34].

Given its large γ value, the 1T‐CrTe_2_/FeTe heterostructure is also a promising candidate for exploring magnetically controllable superconducting diode effect [22]. Moreover, our work demonstrates that superconductivity in FeTe‐based heterostructures persists even when the top‐layer material is ferromagnetic, opening the possibility of exploiting intrinsic magnetism to break time‐reversal symmetry at the interface. In principle, this could enable a superconducting diode effect without an external magnetic field [16, 17, 18]. However, as the magnetic easy axis of CrTe_2_ is out‐of‐plane rather than in‐plane (Figure S18), an in‐plane magnetic field is still required to realize nonreciprocal transport. This constraint can be removed by replacing the top CrTe_2_ layer with a ferromagnetic material with an in‐plane magnetic easy axis.

## 2 Methods

### 2.1 MBE Growth

The 1T‐CrTe_2_/FeTe heterostructures used in this work are grown on insulating 0.5 mm thick SrTiO_3_(100) substrates in a commercial MBE chamber (ScientaOmicron Lab10) with the vacuum better than ∼2 × 10^−10^ mbar. The SrTiO_3_(100) substrates are first soaked in ∼80°C deionized water for ∼2 h and then diluted hydrochloric acid solution (∼4.5% w/w) for ∼2 h. Next, these SrTiO_3_(100) substrates are annealed in a tube furnace with flowing high‐purity oxygen gas at ∼974°C for ∼3 h. These heat treatments make the SrTiO_3_(100) surface passivated, suitable for the MBE growth of 1T‐CrTe_2_/FeTe heterostructures. The heat‐treated SrTiO_3_ (100) substrates are loaded into the MBE chamber and outgassed at ∼600°C for ∼1 h before the MBE growth. High‐purity Fe (99.995%), Te (99.9999%), and Cr (99.999%) are evaporated from Knudsen effusion cells. The growth temperature is ∼340°C for FeTe and ∼ 300°C for 1T‐CrTe_2_. The growth rate is ∼0.3 UC per minute for the FeTe layer and ∼0.25 TL per minute for the 1T‐CrTe_2_ layer, calibrated by AFM and STEM measurements. No capping layer is involved in our measurements.

### 2.2 XRD Measurements

The high‐resolution XRD measurements are carried out at room temperature using a Malvern Panalytical X'Pert3 MRD with Cu‐*K*_α1_ radiation (wavelength λ∼1.5405980 Å).

### 2.3 HAADF‐STEM Measurements

The Aberration‐corrected HAADF‐STEM measurements are performed on an FEI Titan [3] G2 operating parameters at an accelerating voltage of ∼300 kV, with a probe convergence angle of ∼30 mrad, a probe current of ∼70 pA, and HAADF detector angles of 52–253 mrad. More STEM images are shown in Figure S3.

### 2.4 Electrical Transport Measurements

The 1T‐CrTe_2_/FeTe heterostructures grown on 2 mm × 10 mm SrTiO_3_ (100) substrates are scratched into a Hall bar geometry using a computer‐controlled probe station. The effective area of the Hall bar device is ∼ 1 mm × 0.5 mm. The electrical contacts are made by pressing indium spheres on the Hall bar. The electrical transport measurements are conducted using two Physical Property Measurement Systems (PPMS, Quantum Design DynaCool, 1.7 K, 9 T/14 T). The excitation current is ∼1 µA for *R_xx_*‐*T* measurements and ∼100 µA for Hall measurements. The magneto‐transport results are symmetrized or anti‐symmetrized to eliminate mutual pick‐up caused by slight geometrical misalignment of the electrodes. Electrical transport measurements under high magnetic fields (>14 T) are conducted in a capacitor‐driven 65 T pulsed magnet at the National High Magnetic Field Laboratory (NHMFL) in Los Alamos. More transport results are shown in Figures S10–S21.

### 2.5 Nonreciprocal Charge Transport Measurements

The nonreciprocal charge transport measurements are performed using a PPMS (Quantum Design DynaCool, 1.7 K, 9 T) with a single‐axis horizontal rotator module (Figure S22), enabling in‐plane angular rotation of the sample with a ∼0.1° precision. The magnetic field is applied within the rotational plane, and thus remains strictly in‐plane relative to the sample surface during rotation. To minimize canting or misalignment, the sample surface is carefully aligned to the rotational plane. A Keithley 6221 source meter injects an a.c. current through the samples, while the first‐ and second‐harmonic voltages are measured by SRS860 lock‐in amplifiers. Unless otherwise specified, the excitation current is fixed at ∼500 µA with a frequency of ∼6.447 Hz for all measurements, and the external in‐plane magnetic field is applied perpendicular to the excitation current. The second‐harmonic resistance has been anti‐symmetrized to eliminate pick‐up signals from the first‐harmonic resistance. The γ values are obtained by conducting linear regressions to the *R* ^2ω^/*R* ^ω^‐μ_0_ *H*_||_ curves.

### 2.6 MFM Measurements

The MFM measurements are performed with a homebuilt Helium‐3 AFM system using commercial piezoresistive cantilevers (spring constant *k* ∼ 3 N/m and resonant frequency *f*_0_ ∼ 44 kHz). The MFM tips are coated with a nominally 100 nm thick Co layer using magnetron sputtering. The MFM results are extracted by Nanonis SPM Controllers (SPECS) with a phase‐locked loop. The MFM signal, the resonant frequency shift of the cantilever, is proportional to the out‐of‐plane stray field gradient generated by the sample. MFM images are acquired on a scanning plane 200–250 nm above the surface using the constant‐height mode. For balanced visualization of the spatial distribution of the MFM signal at each temperature (i.e., the frequency shift *df*), the color scale is centered at the average MFM signal of each image, with the full color scale mapping to the relative variation of the MFM signal in each image. In situ two‐terminal resistance is measured together with the MFM measurements. To eliminate the electrical contact and lead wire resistance, the residual resistance at *T* = 2 K has been subtracted from the *R*‐*T* curve (Figure 3b). The MFM contrast in Figure 3b and Figure S7d is three times the RMS value of each MFM image, with the value at *T* = 14 K used as an offset to remove the finite value contributed by noise and background.

## Author Contributions

C.‐Z.C. and W.W. conceived and designed the experiment. Z.‐J.Y. and W.Y. performed the MBE growth. Z.‐J.Y., W.Y., A.G.W., Z.W., L.‐J.Z., H.R., and D.Z. performed electrical transport measurements. Y.‐T.C. and W.W. performed the MFM measurements. Z.‐J.Y. performed AFM and XRD measurements. J.S., L.E.W., H.Y., and Z.‐J.Y. performed the electrical transport measurements under high magnetic fields. K.W., H.Y., and Z.‐J.Y. carried out the STEM measurements. Z.‐J.Y., Y.‐T.C., W.W., and C.‐Z.C. analyzed the data and wrote the manuscript with input from all authors.

## Conflicts of Interest

The authors declare no conflicts of interest.

## Supporting information




**Supporting File**: adma72452‐sup‐0001‐SuppMat.pdf.

## Data Availability

The data that support the findings of this study are openly available in [Zenodo] at [https://doi.org/10.5281/zenodo.18073273], reference number [51].
