# Supplementary material for: Meissner Effect and Nonreciprocal Charge Transport in Non‐Topological 1T‐CrTe2/FeTe Heterostructures
Source: Adv Mater. 2026 Feb 5;38(14):e20598. doi: 10.1002/adma.202520598 (PMC12966969; doi:10.1002/adma.202520598)
Supplement: Supplementary file 1 — Supporting File: adma72452‐sup‐0001‐SuppMat.pdf. [file ADMA-38-e20598-s001.pdf]

Supporting Information

Meissner Effect and Nonreciprocal Charge Transport in Non-Topological 1T-$CrTe_2$/FeTe Heterostructures

Zi-Jie Yan[1,5], Ying-Ting Chan[2,5], Wei Yuan[1], Annie G. Wang[1], Hemian Yi[1], Zihao Wang[1], Lingjie Zhou[1], Hongtao Rong[1], Deyi Zhuo[1], Ke Wang[3], John Singleton[4], Laurel E. Winter[4], Weida Wu[2*], and Cui-Zu Chang[1,3*]

[1]Department of Physics, The Pennsylvania State University, University Park, PA 16802, USA

[2]Department of Physics and Astronomy, Rutgers University, Piscataway, NJ 08854, USA

[3]Materials Research Institute, The Pennsylvania State University, University Park, PA 16802, USA

[4]National High Magnetic Field Laboratory, Los Alamos, NM 87544, USA

[5]These authors contributed equally: Zi-Jie Yan and Ying-Ting Chan

[*]Corresponding authors: wdwu@physics.rutgers.edu (W.W.); cxc955@psu.edu (C.-Z. C.)

Content:

I. **Supplementary Figures**

II. **Supplementary Tables**

III. **Supplementary Text**

1. **More discussion on interfaced-induced superconductivity in 1T-$CrTe_2$/FeTe**
2. **Derivation of the nonreciprocal charge transport**
3. **Magnetic anisotropy of 1T-$CrTe_2$ and its influence on the nonreciprocal transport**
4. **Thickness-dependent nonreciprocal transport in 1T-$CrTe_2$/FeTe**
5. **Role of self-heating-induced thermal gradient in $R^{2\omega}$**
6. **Absence of the d.c. diode effect in 1T-$CrTe_2$/FeTe**
7. **Discussion on the superconducting mechanism of FeTe-based heterostructures**
8. **Discussion on the a.c. frequency dependence of the nonreciprocal transport**

References

I. Supplementary Figures

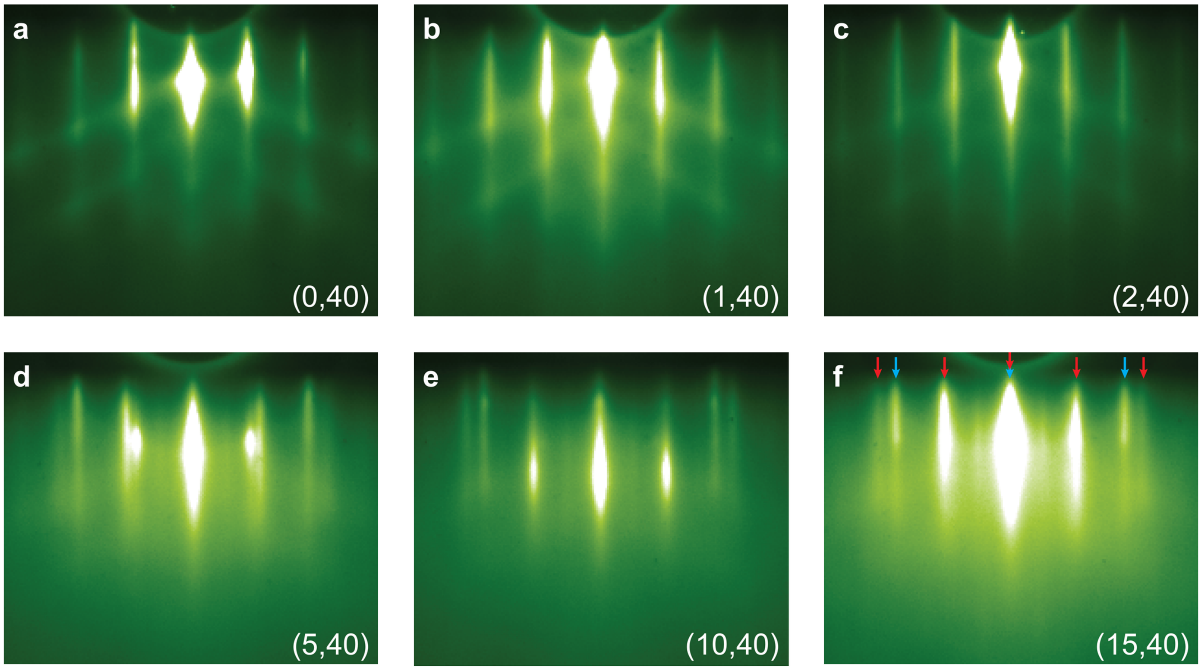

Figure S1| RHEED patterns of the (*m*, 40) heterostructures. **a**, $m = 0$, **b**, $m = 1$, **c**, $m = 2$, **d**, $m = 5$, **e**, $m = 10$, and **f**, $m = 15$. The red (blue) arrows in (**f**) mark the diffraction patterns along the $[11\bar{2}0]$ ($[1\bar{1}00]$) direction of the $CrTe_2$ layer, indicating the twin-domain structure of the $CrTe_2$ layer. As *m* increases, the RHEED patterns of the FeTe layer first become elongated (**b**, **c**) and then gradually replaced by the RHEED patterns of the $CrTe_2$ layer (**d**-**f**).

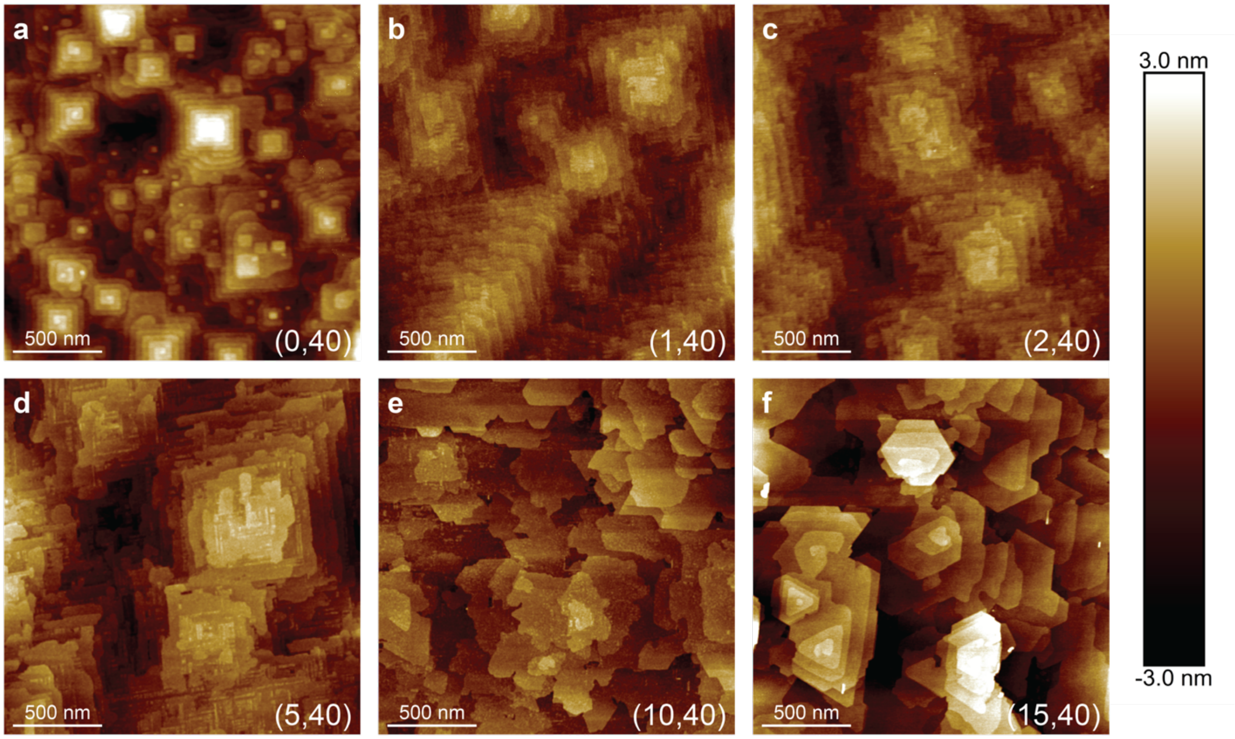

Figure S2| AFM images of the (*m*, 40) heterostructures. **a**, $m = 0$, **b**, $m = 1$, **c**, $m = 2$, **d**, $m = 5$, **e**, $m = 10$, and **f**, $m = 15$. As m increases, the $CrTe_2$ TL initially grows from the edges of the FeTe square-shaped islands (**b-d**) and eventually covers the entire FeTe layer, forming triangular-shaped islands (**e**, **f**).

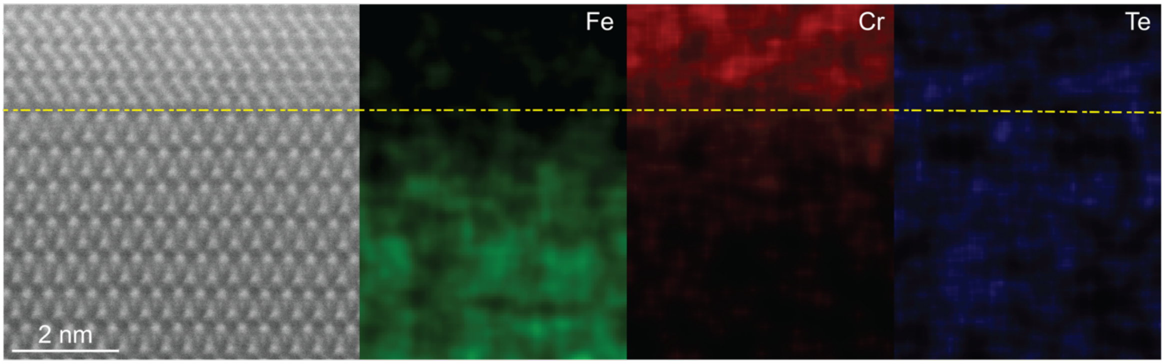

Figure S3| Cross-sectional STEM image and EDS maps of the (15, 40) heterostructure. The yellow dashed line indicates the sharp interface between 1T-$CrTe_2$ and FeTe. This image is taken with the sample rotated by 15° relative to the STEM image shown in Fig. 1e.

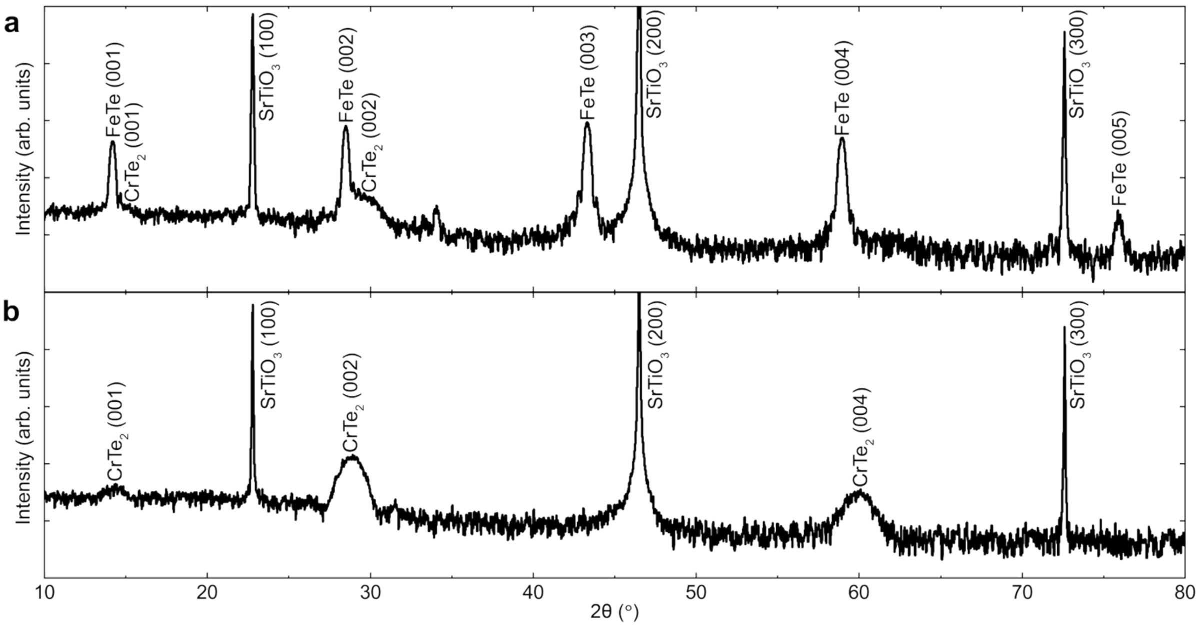

Figure S4| XRD spectra of the (15, *n*) heterostructures grown on heat-treated $SrTiO_3$(100). **a**, n = 40, **b**, n = 0. Because of the similar c-axis lattice constant of the 1T-$CrTe_2$ and FeTe layers, the diffraction peaks of the 1T-$CrTe_2$ layer overlap with those of the FeTe layer in (**a**).

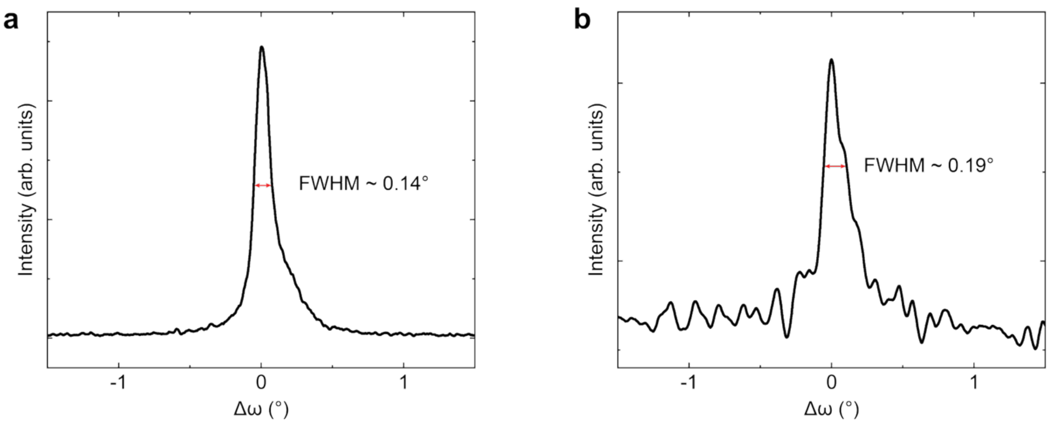

Figure S5| Rocking curves of the (15, 40) heterostructure. a, **b**, Rocking curves of FeTe (003) diffraction peak (**a**) and $CrTe_2$ (002) diffraction peak (**b**).

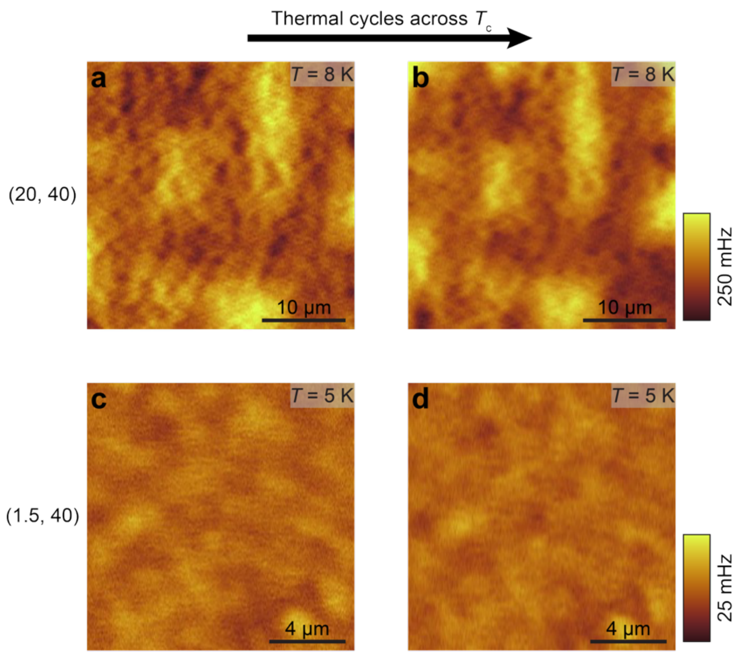

Figure S6| MFM images of 1T-$CrTe_2$/FeTe heterostructures with (*m*, *n*) = (20, 40) and (1.5, 40) before and after thermal cycling across their superconducting T_c. a, **b**, MFM images of the (20, 40) heterostructure measured at T = 8 K before (**a**) and after (**b**) multiple thermal cycles across its superconducting T_c. **c**, **d**, MFM images of the (1.5, 40) heterostructure measured at T = 5 K before (**c**) and after (**d**) a single thermal cycle across its superconducting T_c. During MFM measurements, the magnetic tip is ~250 nm (**a**, **b**) or ~200 nm (**c**, **d**) above the sample surface, and an external magnetic field of ~0.2 T is applied. For MFM images in (**a-d**), the color scale is centered at the average MFM signal of each image, with the full color scale mapping to the relative variation of the MFM signal in each image (Methods).

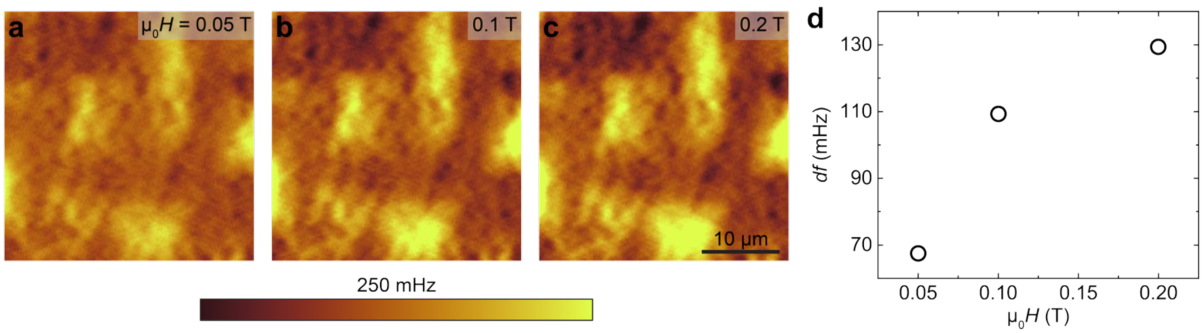

Figure S7| MFM images of the (20, 40) heterostructure under different magnetic fields measured at T = 8 K. a-c, MFM images measured at $\mu_0 H$ = 0.05 T (**a**), 0.1 T (**b**), and 0.2 T (**c**). **d,** $\mu_0 H$ dependence of the MFM contrast *df* at T = 8 K. During MFM measurements, the magnetic tip is ~250 nm above the sample surface, and an external magnetic field of ~0.2 T is applied. For MFM images in (**a**-**c**), the color scale is centered at the average MFM signal of each image, with the full color scale mapping to the relative variation of the MFM signal in each image (Methods). The MFM contrast is defined as three times of the root mean square (RMS) value of each MFM image in (**a**-**c**) (Methods).

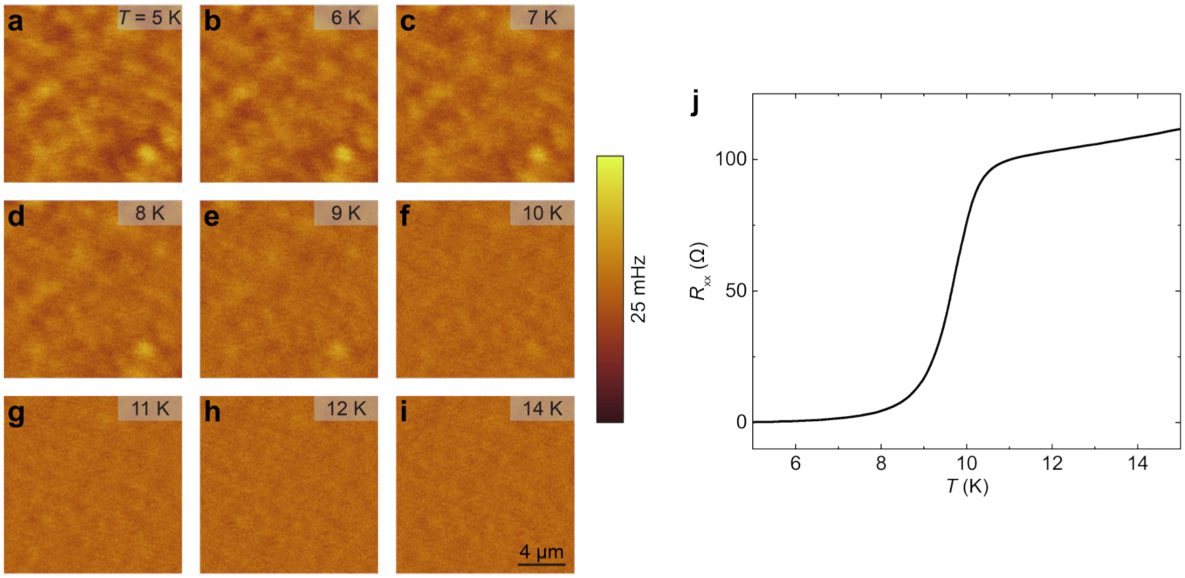

Figure S8| Meissner effect in the (1.5, 40) heterostructure. a-i, MFM images of the (1.5, 40) heterostructure measured at T = 5 K (**a**), 6 K (**b**), 7 K (**c**), 8 K (**d**), 9 K (**e**), 10 K (**f**), 11 K (**g**), 12 K (**h**), and 14 K (**i**). During MFM measurements, the magnetic tip is ~200 nm above the sample surface, and an external magnetic field of ~0.2 T is applied. For MFM images in (**a-d**), the color scale is centered at the average MFM signal of each image, with the full color scale mapping to the relative variation of the MFM signal in each image (Methods). **j**, T-dependent R_{xx} of the (1.5, 40) heterostructure.

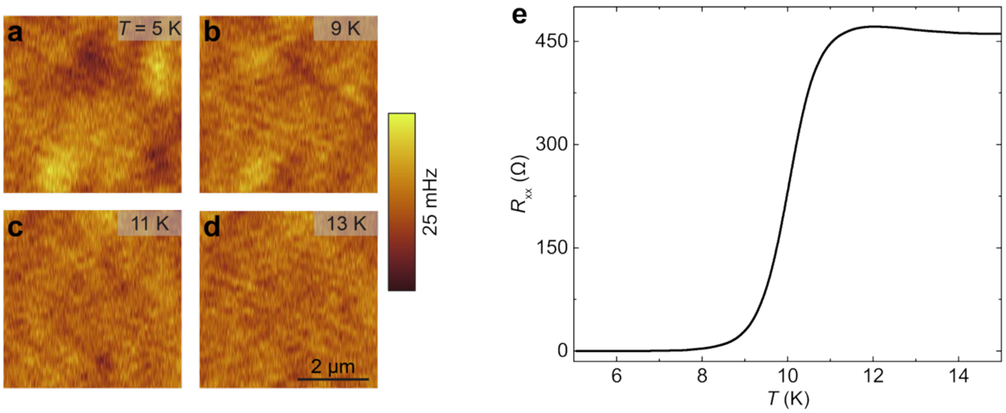

Figure S9| Meissner effect in the (10, 10) heterostructure. a-d, MFM images of the (10, 10) heterostructure measured at T = 5 K (**a**), 9 K (**b**), 11 K (**c**), and 13 K (**d**). During MFM measurements, the magnetic tip is ~200 nm above the sample surface, and an external magnetic field of ~0.2 T is applied. For MFM images in (**a-d**), the color scale is centered at the average MFM signal of each image, with the full color scale mapping to the relative variation of the MFM signal in each image (Methods). **e**, T-dependent R_{xx} of the (10, 10) heterostructure. (e) is reused from Fig. 2b of the main text.

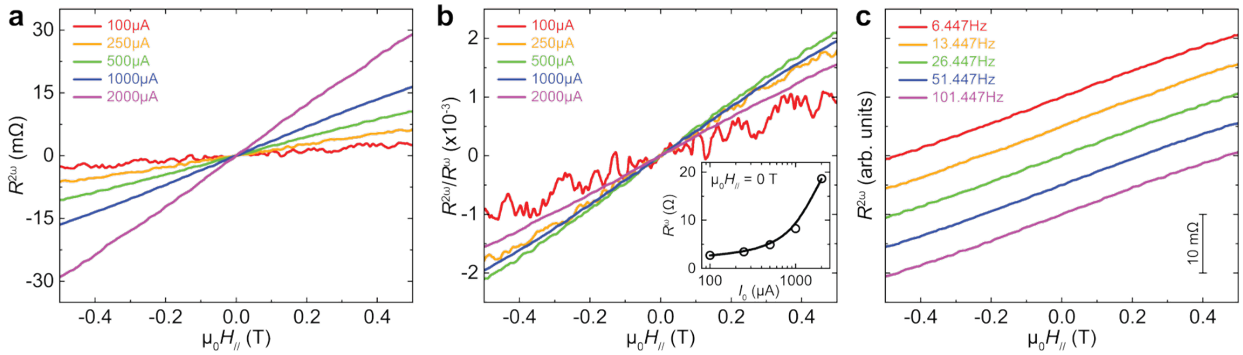

Figure S10| Nonreciprocal charge transport measurements on the (10, 20) heterostructure under different excitation currents and frequencies at T = 11.6 K. a, $\mu_0 H_{||}$ dependence of the second-harmonic resistance $R^{2\omega}$ under different excitation currents. **b**, $\mu_0 H_{||}$ dependence of the ratio between second- and first-harmonic resistances $R^{2\omega}/R^{\omega}$ under different excitation currents. Inset: the excitation current dependence of R^{ω} at $\mu_0 H_{||} = 0$ T. **c**, $\mu_0 H_{||}$ dependence of $R^{2\omega}$ under different frequencies. Each curve is shifted by 5 mΩ.

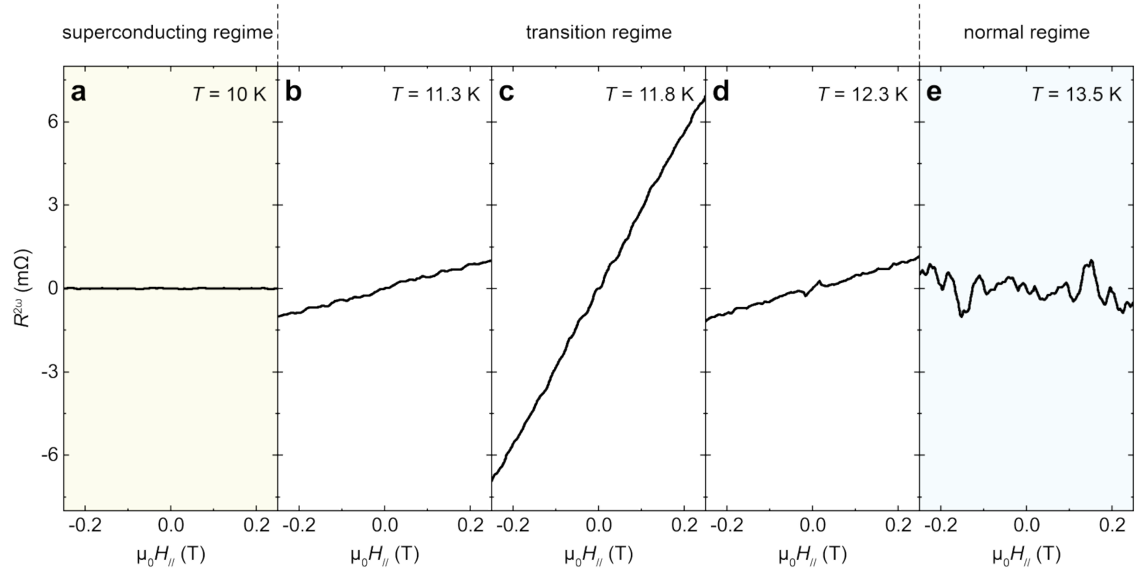

Figure S11| Second-harmonic resistance $R^{2\omega}$ of the (10, 20) heterostructure measured at different temperatures. **a**, $T = 10$ K, **b**, $T = 11.3$ K, **c**, $T = 11.8$ K, **d**, $T = 12.3$ K, **e**, $T = 13.5$ K. The nonreciprocal charge transport behavior is observed only during the superconducting transition regime (**b**-**d**).

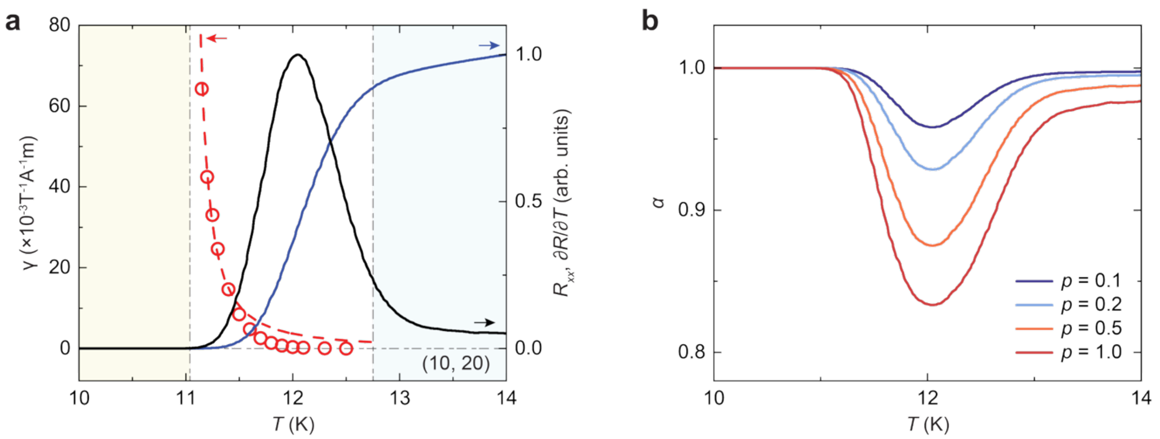

Figure S12| Evaluation of the self-heating contribution to the observed nonreciprocal transport in the (10, 20) heterostructure. a, T dependence of the magneto-chiral anisotropy coefficient γ (red circles), the sheet longitudinal resistance R_{xx} (blue curve), and the first-order derivative of R_{xx} (i.e., $\partial R^{\omega}/\partial T$, black curve). The red dashed curve is fitted by the formula $\gamma = \beta(T - T_{\mathrm{BKT}})^{-1.5}$, where T_{BKT} is the BKT superconducting transition temperature, and β is a fitting coefficient. **b**, T dependence of the factor α, calculated with different p values. The red circles and blue curves in (**a**) are reused from Fig. 4c of the main text.

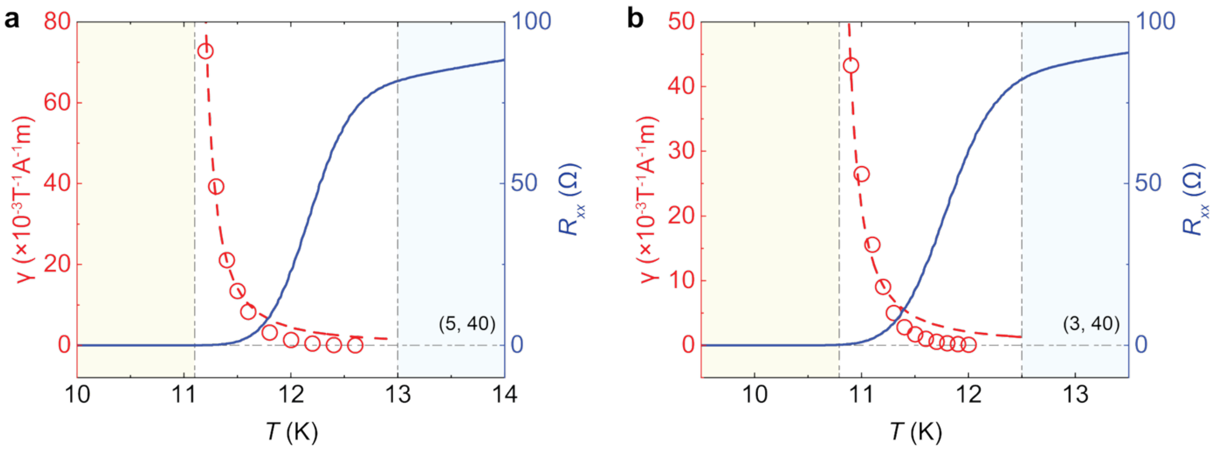

Figure S13| Nonreciprocal charge transport in more 1T-$CrTe_2$/FeTe heterostructures. a, b, *T* dependence of magneto-chiral anisotropy coefficient γ (red circles) and sheet resistance R_{xx} (blue curve) in the (5, 40) (**a**) and (3, 40) (**b**) heterostructures. The large nonreciprocal charge transport occurs only during the superconducting transition regime. The γ values of the (5, 40) and (3, 40) heterostructures are 72.8×10^{-3} $T^{-1}\cdot A^{-1}m$ at $T = 11.2$ K and 43.3×10^{-3} $T^{-1}\cdot A^{-1}m$ at $T = 10.9$ K, respectively.

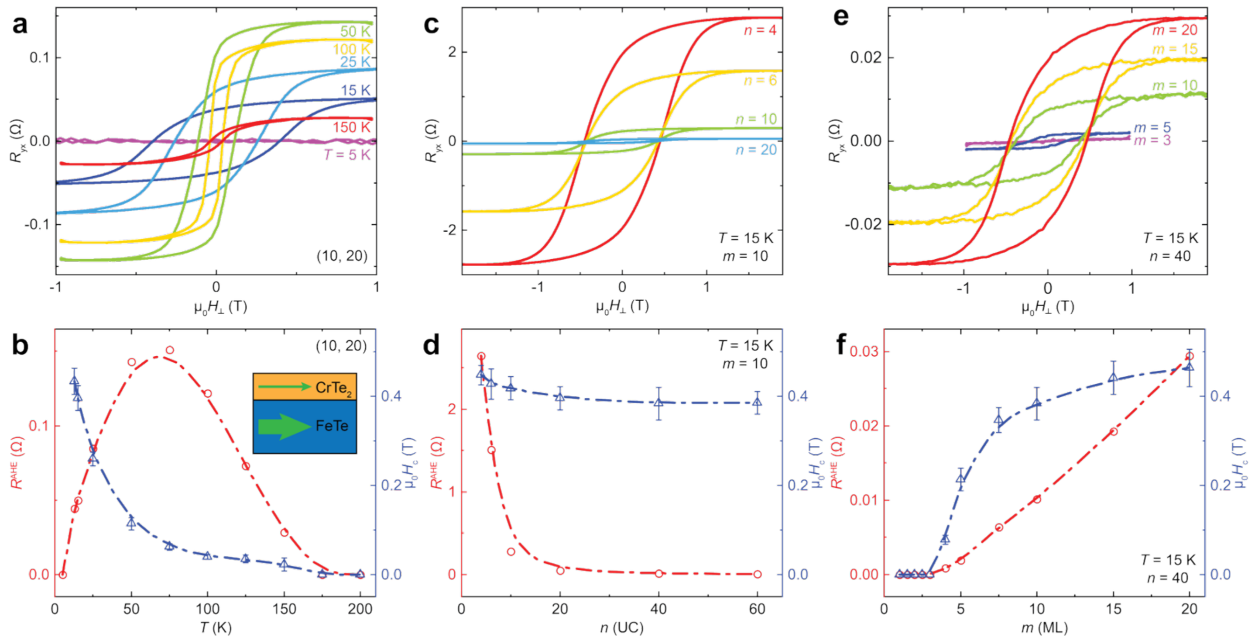

Figure S14| Ferromagnetic properties of the (*m*, *n*) heterostructures. **a**, Ferromagnetic hysteresis loops of the (10, 20) heterostructure measured at different *T*. **b**, *T* dependent R^{AHE} (red) and $\mu_0 H_c$ (blue) of the (10, 20) heterostructure. Inset: Schematic of the current flow through the $CrTe_2$ and FeTe layers. The arrow size indicates the magnitude of the current in each layer. **c**, Ferromagnetic hysteresis loops of the (10, *n*) heterostructures measured at *T* = 15 K. **d**, *n* dependent R^{AHE} (red) and $\mu_0 H_c$ (blue) of the (10, *n*) heterostructures. **e**, Ferromagnetic hysteresis loops of the (*m*, 40) heterostructures measured at *T* = 15 K. **f**, *m* dependent of R^{AHE} (red) and $\mu_0 H_c$ (blue) of the (*m*, 40) heterostructures.

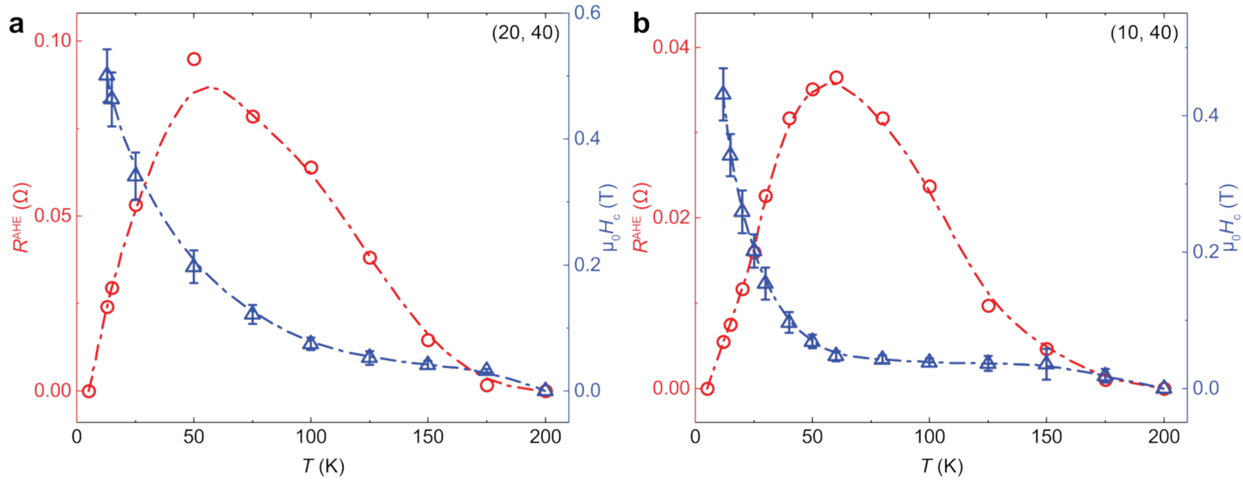

Figure S15| Temperature evolution of ferromagnetism in the (*m*, *n*) heterostructures. a, b, *T* dependent R^{AHE} (red) and μ_0H_c (blue) of the (20, 40) (**a**) and (10, 40) (**b**) heterostructures.

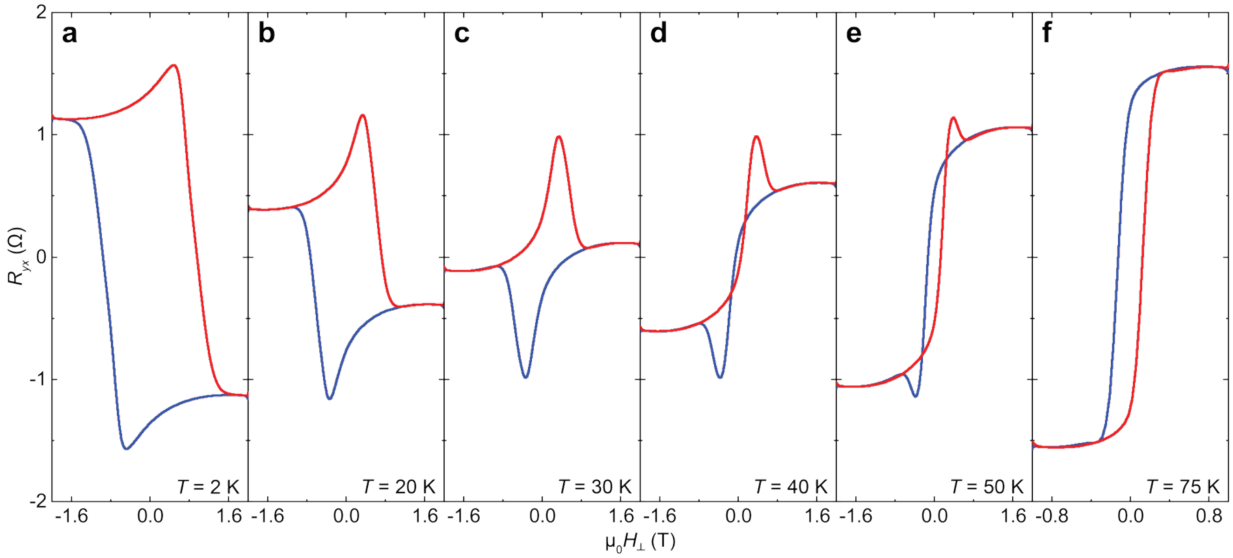

Figure S16| Hall traces of 15 TL 1T-$CrTe_2$/$SrTiO_3$(100). a-f, $\mu_0 H_\perp$ dependence of the Hall resistance R_{yx} measured at $T = 2$ K (**a**), $T = 20$ K (**b**), $T = 30$ K (**c**), $T = 40$ K (**d**), $T = 50$ K (**e**), and $T = 75$ K (**f**). As T increases, a sign reversal of R_{yx} and a topological Hall effect are observed, consistent with prior studies[1,2].

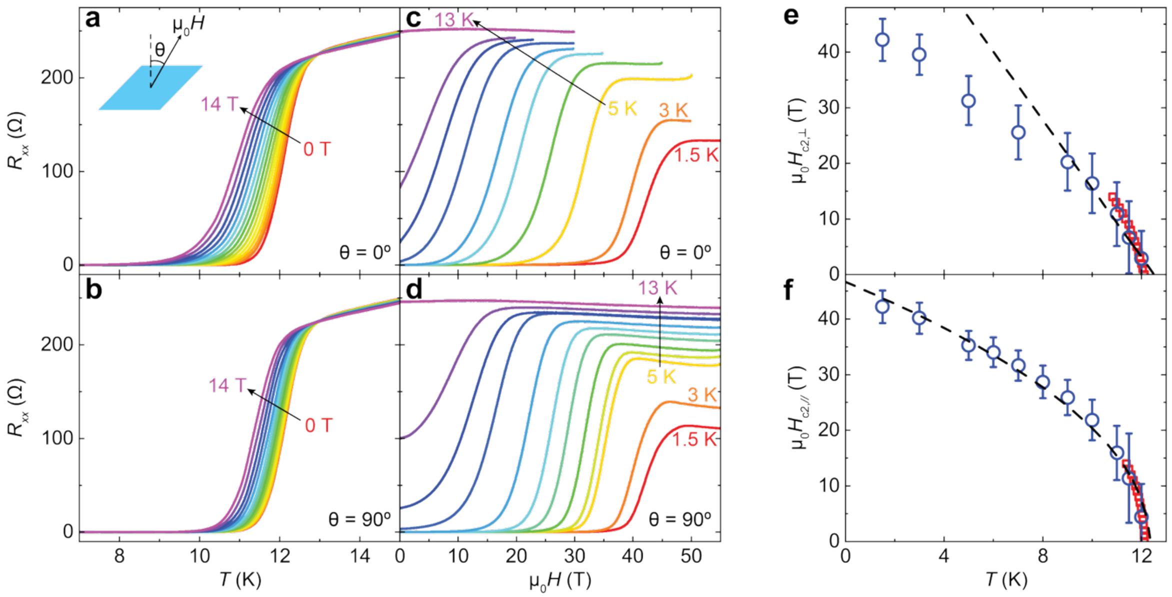

Figure S17| Magnetoresistance of the (10,15) heterostructure. a, **b**, R_{xx}-T curves under different $\mu_0 H$. (**a**) $\theta = 0^{\text{o}}$ (i.e., out-of-plane) and (**b**) $\theta = 90^{\text{o}}$ (i.e., in-plane). **c**, **d**, R_{xx}-$\mu_0 H$ curves under different T. (**c**) $\theta = 0^{\text{o}}$ (i.e., out-of-plane) and (**d**) $\theta = 90^{\text{o}}$ (i.e., in-plane). **e**, **f**, T dependence of $\mu_0 H_{c2,\perp}$ (**e**) and $\mu_0 H_{c2,||}$ (**f**). The red data points are extracted from (**a**, **b**), while the blue data points are extracted from (**c**, **d**). The error bars for the blue data points are estimated by the magnetic field difference between the normal state and the zero-resistance state. The black dashed lines in (**e**, **f**) are the curves fitted using Ginzburg-Landau (G-L) theory in the 2D limit. The effective superconducting layer thickness d_{sc} and the Ginzburg-Landau coherence length ξ_{GL} are estimated to be ~11.81 nm and ~2.07 nm, respectively, for the (10, 15) heterostructure.

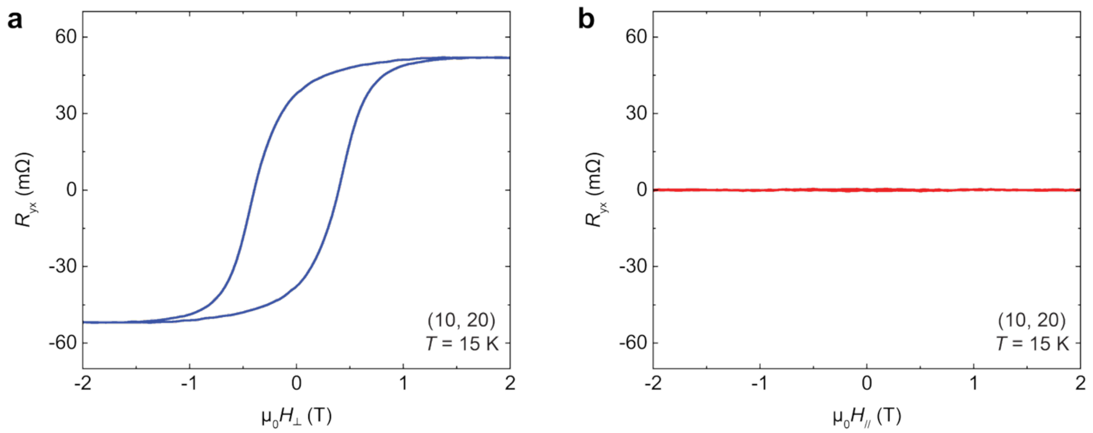

Figure S18| Magneto-transport results of the (10, 20) heterostructure measured at *T* = 15 K. **a**, **b**, R_{yx} measured under out-of-plane (**a**) and in-plane (**b**) magnetic fields.

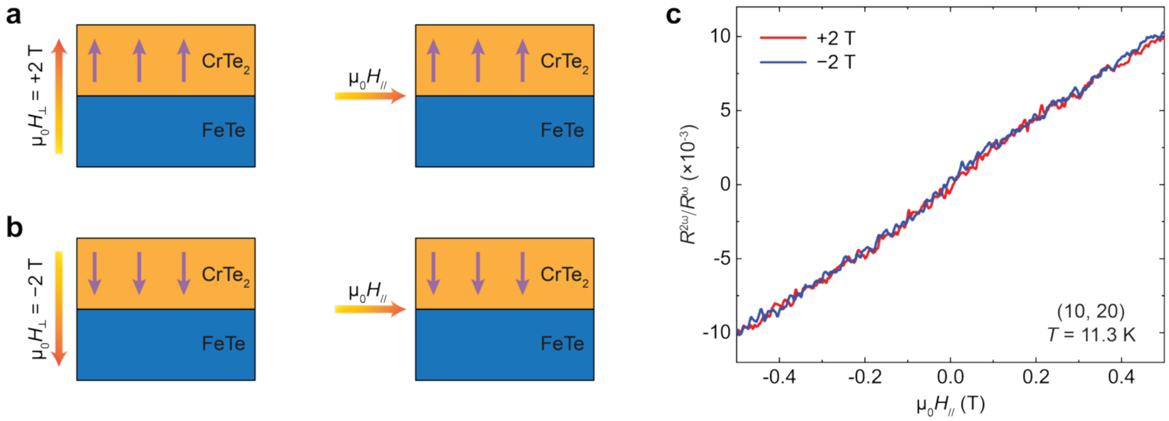

Figure S19| Nonreciprocal charge transport in the (10, 20) heterostructure with oppositely oriented magnetizations of the $CrTe_2$ layer. a, b, Schematics of the nonreciprocal transport with the magnetization of the $CrTe_2$ layer pointing upward (**a**) and downward (**b**), respectively. Before performing nonreciprocal transport measurements with an in-plane magnetic field $\mu_0 H_{\|}$, an out-of-plane magnetic field $\mu_0 H_\perp$ of +2 T (**a**) or −2 T (**b**) is used to train the magnetization of the top 10 TL $CrTe_2$ layer in opposite directions in our experiments. After magnetization training, $\mu_0 H_\perp$ is withdrawn. **c,** $\mu_0 H_{\|}$ dependence of second-harmonic response (i.e., $R^{2\omega}/R^{\omega}$) measured at T = 11.3 K with opposite magnetizations of the $CrTe_2$ layer, trained by $\mu_0 H_\perp$ = +2 T (red) and −2 T (blue), respectively.

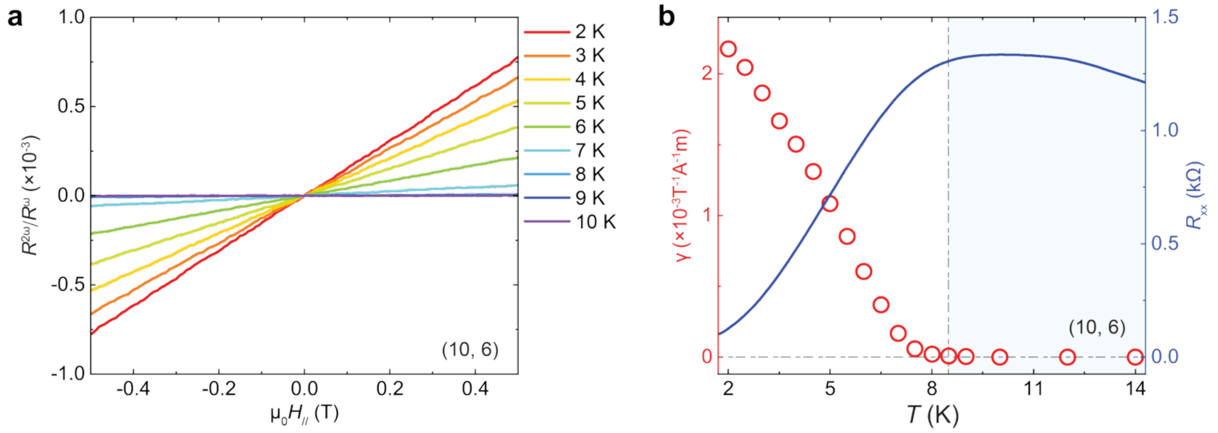

Figure S20| Nonreciprocal charge transport in the (10, 6) heterostructure. a, $\mu_0 H_{\parallel}$ dependence of second-harmonic response (i.e., $R^{2\omega}/R^{\omega}$) measured at different temperatures. **b**, T dependence of the magneto-chiral anisotropy coefficient γ (red circles) and the sheet longitudinal resistance R_{xx} (blue curve). The γ value is ~2.18×10^{-3} $T^{-1}\cdot A^{-1}m$ at $T = 2$ K for the (10, 6) heterostructure

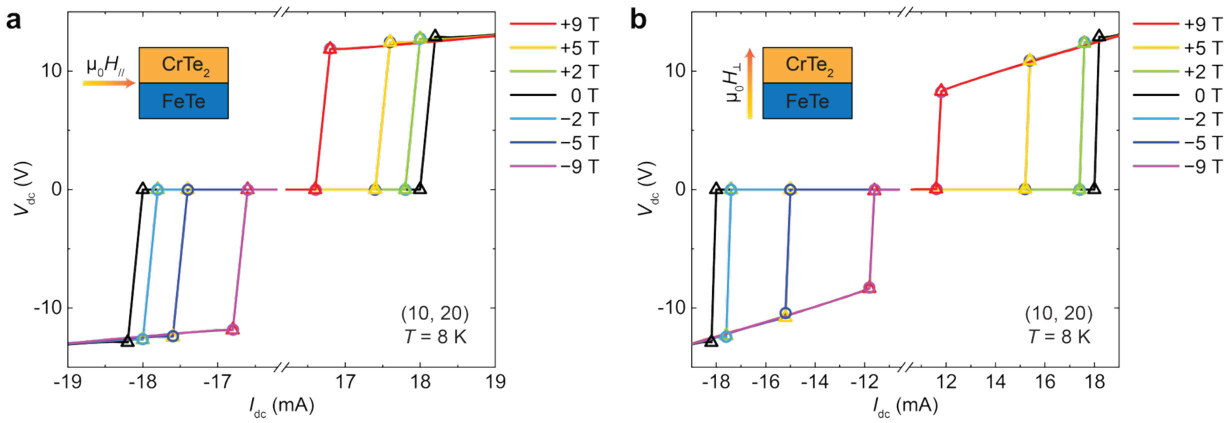

Figure S21| Absence of the d.c. superconducting diode effect in the (10, 20) heterostructure Hall bar device. a, **b**, *I*-*V* curves measured under different in-plane (**a**) and out-of-plane (**b**) magnetic fields at T = 8 K. The triangles and circles highlight the data points near the critical currents I_c in the *I*-*V* curves measured at positive and negative magnetic fields, respectively, which match well with each other. Inset: Schematic of the measurement configurations.

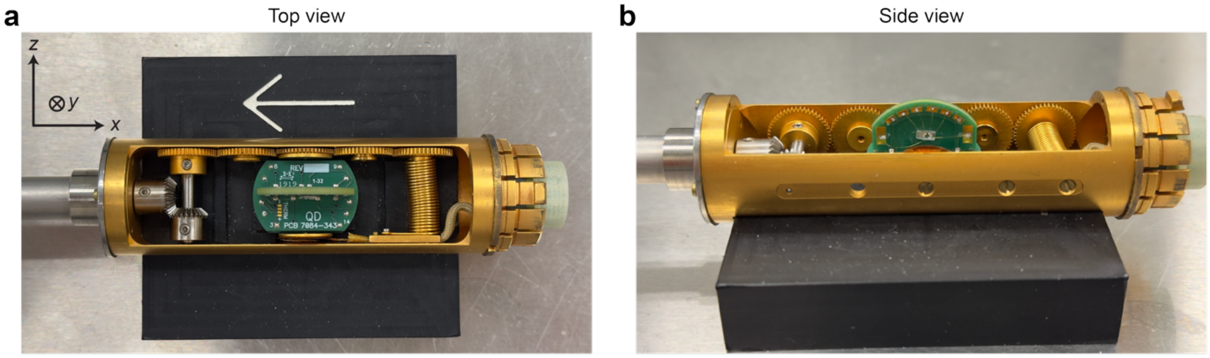

Figure S22| Optical images of the horizontal rotator module of the Quantum Design DynaCool PPMS. **a**, **b**, Top view (**a**) and side view (**b**) of the horizontal rotator module. The green part is the measurement chip, on which the $CrTe_2$/FeTe Hall bar device is mounted. The magnetic field is applied along the white arrow direction in (**a**). With this measurement chip, the rotation of the sample is confined to the *x*-*y* plane, which is parallel to the applied magnetic field, enabling the in-plane magnetic field dependence measurements in Fig. 4b inset of the main text.

II. Supplementary Tables

Table S1| Lattice constants of FeTe and its partner layers

Materials	Lattice structure	In-plane lattice constant	References
Bi_2Te_3	Trigonal	4.386 Å	Ref.[3]
Sb_2Te_3	Trigonal	4.2423 Å	Ref.[4]
1T-$CrTe_2$	Trigonal	3.93 Å	Ref.[2]
FeTe	Tetragonal	3.8220 Å	Ref.[5]
$MnBi_2Te_4$	Trigonal	4.3 Å	Ref.[6]

Table S2| Work functions of FeTe and its partner layers

Materials	Work functions	References
Bi_2Te_3	5.3 eV	Ref.[7]
Sb_2Te_3	5.0 eV	Ref.[7]
1T-$CrTe_2$	4.7~5.0 eV	JARVIS-DFT Database (NIST) (Ref.[8])
FeTe	4.4~4.8 eV	Ref.[7]
$MnBi_2Te_4$	4.0~4.4 eV	Ref.[9]

III. Supplementary Text

1. More discussion on interfaced-induced superconductivity in 1T-$CrTe_2$/FeTe

In this section, we first clarify one common misunderstanding of interfacial superconductivity. The term "interfacial superconductor" does not imply that superconductivity is strictly limited to the interface between two adjacent mono-atomic layers. Depending on the depairing potential strength, the interfacial superconductivity could extend many atomic layers into adjacent materials. So far, all discovered interfacial superconductors usually have a critical thickness for both component layers. For the well-studied $LaAlO_3$/$SrTiO_3$ system, the $SrTiO_3$ is usually a bulk crystal, and the superconductivity emerges only when $LaAlO_3$ is greater than ~3 UC (Ref.[10]). For all MBE-grown copper-oxide-based interfacial superconductors, e.g., $La_{1.55}Sr_{0.45}CuO_4$/La_2CuO_4, the thickness of the bottom insulating La_2CuO_4 layer is ~40 UC, and the superconductivity emerges only when the thickness of the top metallic $La_{1.55}Sr_{0.45}CuO_4$ is greater than ~3 UC (Ref.[11]).

In our experiments, the as-grown FeTe films are antiferromagnetic and non-superconducting, with superconductivity emerging only after the deposition of the $CrTe_2$ layer (Fig. 2a,c). This observation indicates that the $CrTe_2$/FeTe interface plays a key role in triggering the superconducting state of our 1T-$CrTe_2$/FeTe heterostructures. We note that while the $CrTe_2$/FeTe interface is essential for inducing superconductivity, the interface-induced superconductivity is not necessarily 2D in our 1T-$CrTe_2$/FeTe heterostructures.

To investigate the role of the 1T-$CrTe_2$/FeTe interface and determine the dimensionality of the induced superconductivity, we perform electrical transport measurements on a (10, 15) heterostructure under in-plane and out-of-plane magnetic fields to construct its phase diagram (Fig. S17). The effective thickness d_{sc} of the superconducting and the Ginzburg-Landau coherence length ξ_{GL} are extracted by fitting the data with the Ginzburg-Landau theory in the 2D limit, where

Φ_0 is the flux quantum.

$$\mu_0 H_{c2,\perp}(T) = \frac{\Phi_0}{2\pi\xi_{GL}^2}(1 - {}^{T}/_{T_c}) \tag{S1}$$

$$\mu_0 H_{c2,\parallel}(T) = \frac{\sqrt{3}\Phi_0}{\pi\xi_{GL} d_{sc}}(1 - {}^{T}/_{T_c})^{\frac{1}{2}} \tag{S2}$$

For the (10, 15) heterostructure, its d_{sc} and ξ_{GL} are fitted to be ~11.81 nm and ~2.07 nm, respectively (Fig. S17e,f). We note that this d_{sc} value is comparable to the effective superconducting thickness in other known interfacial superconductors. For the aforementioned $LaAlO_3/SrTiO_3$ system, the value of d_{sc} is ~10 nm even if both $LaAlO_3$ and $SrTiO_3$ are large-gap insulators[12]. In addition, for the recently discovered interfacial superconductivity in $EuO/KTaO_3$ heterostructures, the value of d_{sc} is ~5.1 nm (Ref.[13]). Therefore, the d_{sc} ~ 11.81 nm is less than the thickness of the (10,15) heterostructure (~15.50 nm), further implying that the superconductivity realized in 1T-$CrTe_2$/FeTe heterostructures stems from an interfacial effect.

For a superconducting thin film in the 2D regime, the effective superconducting thickness must be much smaller than its coherent length, i.e., $d_{sc} \ll \xi_{GL}$ (Refs.[14,15]), and the 2D limit breaks down once $d_{sc} > \xi_{GL}$ with a commonly used crossover criteria $d_{sc} \approx 1.84\xi_{GL}$ (Ref.[16]). As noted above, the the superconducting region thickness d_{sc} of the $CrTe_2$/FeTe heterostructure is ~11.81 nm, which substantially exceeds its coherence length ξ_{GL} ~ 2.07 nm, the interface-induced superconductivity here is in the 3D regime rather than the 2D thin-film limit. In this regime, the behavior of $\mu_0 H_{c2}$ deviates from the 2D Ginzburg–Landau theory at low temperatures, leading to the absence of the angular anisotropy characteristic on $\mu_0 H_{c2,\perp}$ and $\mu_0 H_{c2,||}$ (i.e., the Tinkham cusp), consistent with the observed nearly isotropic upper critical magnetic fields $\mu_0 H_{c2}$ at low temperatures (Fig. S17e,f). These deviations may reflect multiband effects[17] and/or high magnetic field Pauli-limiting behavior[18]. We note that similar features have also been observed in prior studies on other FeTe-

based heterostructures[7,19-22]. These observations indicate that the superconducting mechanism across FeTe-based heterostructures likely originate from the underlying FeTe layer, as discussed in main text.

2. Derivation of the nonreciprocal charge transport

From Eq. 1, an a.c. current $I = \sqrt{2}I_0 \sin(\omega t)$ can generate an a.c. voltage $V_{xx} = R^{\omega} I + \gamma R^{\omega}\left[(\mu_0 \overrightarrow{H_{\parallel}} \times \hat{z}) \cdot \vec{I}\right] I$ in the 1T-$CrTe_2$/FeTe heterostructure. Under an in-plane magnetic field $\mu_0 \overrightarrow{H_{\parallel}}$ (Fig. 4a), the longitudinal voltage V_{xx} can be calculated by substituting I by $\sqrt{2}I_0 \sin(\omega t)$.

$$\begin{aligned} V_{xx} &= R^{\omega}\sqrt{2}I_0 \sin(\omega t) + \gamma R^{\omega} \mu_0 H_{\parallel} \cos\phi \sqrt{2}I_0 \sin(\omega t) \sqrt{2}I_0 \sin(\omega t) \\ &= R^{\omega}\sqrt{2}I_0 \sin(\omega t) + 2\gamma R^{\omega} \mu_0 H_{\parallel} \cos\phi \, {I_0}^2 \sin^2(\omega t) \\ &= R^{\omega}\sqrt{2}I_0 \sin(\omega t) + 2\gamma R^{\omega} \mu_0 H_{\parallel} \cos\phi \, {I_0}^2 \frac{1 - \cos(2\omega t)}{2} \\ &= \gamma R^{\omega} B \cos\phi \, {I_0}^2 + \sqrt{2}R^{\omega} I_0 \sin(\omega t) - \gamma R^{\omega} \mu_0 H_{\parallel} \cos\phi \, {I_0}^2 \cos(2\omega t) \\ &= \gamma R^{\omega} B \cos\phi \, {I_0}^2 + \sqrt{2}R^{\omega} I_0 \sin(\omega t) + \gamma R^{\omega} \mu_0 H_{\parallel} \cos\phi \, {I_0}^2 \sin\left(2\omega t - \frac{\pi}{2}\right) \end{aligned} \quad \text{(S3)}$$

Based on Eq. S3, the second term corresponds to the first-harmonic response $R^{\omega} = \frac{\sqrt{2}R^{\omega} I_0}{\sqrt{2}I_0} = R^{\omega}$, the third term is the second-harmonic response $R^{2\omega} = \frac{\gamma R^{\omega} \mu_0 H_{\parallel} \cos\phi {I_0}^2}{\sqrt{2}I_0} = \frac{\gamma R^{\omega} \mu_0 H_{\parallel} \cos\phi I_0}{\sqrt{2}}$. Therefore, the magneto-chiral anisotropy coefficient $\gamma = \frac{\sqrt{2}}{\cos\phi I_0} \frac{R^{2\omega}/R^{\omega}}{\mu_0 H_{\parallel}}$, where $\frac{R^{2\omega}/R^{\omega}}{\mu_0 H_{\parallel}}$ is achieved by a linear fit to the $R^{2\omega}/R^{\omega} - \mu_0 H_{\parallel}$ curves.

3. Magnetic anisotropy of 1T-$CrTe_2$ and its influence on the nonreciprocal transport

We perform systematic magneto-transport measurements on 1T-$CrTe_2$/FeTe heterostructures with varying (m, n) under out-of-plane magnetic fields (Figs. S14 and S15). Our results demonstrate that 1T-$CrTe_2$ films grown on FeTe exhibit a perpendicular magnetic anisotropy, in good agreement with prior studies on MBE-grown 1T-$CrTe_2$ films[1,2,23].

In our nonreciprocal transport measurements, an in-plane magnetic field $\mu_0 H_{||}$ of up to 0.5 T is applied. To ensure this in-plane magnetic field does not alter the magnetization orientation of the 1T-$CrTe_2$ layer, we perform magneto-transport measurements on the (10, 20) heterostructure under an in-plane magnetic field. At T = 15 K, near the superconducting transition regime where we perform the nonreciprocal charge transport measurements, no discernible magnetic hysteresis loop is observed under in-plane magnetic field up to ±2 T (Fig. S18b). This observation confirms that the 0.5 T in-plane magnetic field used in our nonreciprocal charge transport measurements does not affect the magnetization orientation of the 1T-$CrTe_2$ films.

To further investigate the influence of the magnetization of the top $CrTe_2$ layer, we performed nonreciprocal transport measurements on the (10, 20) heterostructure with the magnetization of the top 10 TL $CrTe_2$ layer pointing upward and downward, respectively (Fig. S19a,b). We found that the $\mu_0 H_{||}$ dependence of second-harmonic response (i.e., $R^{2\omega}/R^{\omega}$) remains nearly identical regardless of the magnetization direction of the top 10 TL $CrTe_2$ layer (Fig. S19c), and no second-harmonic response has been observed at zero magnetic field. Therefore, the nonreciprocal transport cannot be realized in our 1T-$CrTe_2$/FeTe heterostructures at zero magnetic field. This observation is also consistent with the perpendicular magnetic anisotropy of $CrTe_2$ films grown on FeTe (Figs. S14 and S18).

4. Thickness dependent nonreciprocal transport in 1T-$CrTe_2$/FeTe

We perform second harmonic transport measurements on 1T-$CrTe_2$/FeTe heterostructures with different (m, n) (Figs. 4, S13, and S20). As discussed in main text, with increasing m or n, the superconducting transition gets sharper and T_c increase and saturates at ~12 K for $m \geq 3$ and $n \geq 15$ (Fig. 2). For 1T-$CrTe_2$/FeTe heterostructures with (m, n) = (10, 20), (5, 40), and (3, 40), which all exhibit a similar saturated T_c ~12 K, the nonreciprocal coefficient γ is comparable near their

$T_{c,0}$ (Figs. 4c and S13): $\gamma = 64.3 \times 10^{-3}$ $T^{-1}\cdot A^{-1}m$ at $T = 11.15$ K for (10, 20); $\gamma = 72.8 \times 10^{-3}$ $T^{-1}\cdot A^{-1}m$ at $T = 11.2$ K for (5, 40); and $\gamma = 43.3 \times 10^{-3}$ $T^{-1}\cdot A^{-1}m$ at $T = 10.9$ K for (3, 40). These thicker samples exhibit similar nonreciprocal transport behaviors with thickness-independent γ values. Although the γ values do not strictly saturate to a constant, the small fluctuation in γ may arise from the sample variations.

In contrast, for the thinner 1T-$CrTe_2$/FeTe heterostructures with weaker superconductivity, i.e., a lower T_c and a broader superconducting transition, a reduced nonreciprocal transport behavior with smaller γ is observed. For the (10, 6) heterostructure with weaker superconductivity, the broader superconducting transition does not reach a zero-resistance state even at $T = 2$ K (Fig. 2b), we observe nonreciprocal transport behaviors (i.e., linear dependence of $R^{2\omega}/R^{\omega}$ on $\mu_0 H_{||}$) over a wide temperature range, i.e., 2 K $\leq T \leq$ 8 K (Fig. S20a), consistent with our observations on thicker samples (Fig. S11). However, unlike the 1T-$CrTe_2$/FeTe heterostructures with a sharper superconducting transition and $T_c \sim 12$ K (Figs. 4c and S13), the γ value is only ~2.18×10^{-3} $T^{-1}\cdot A^{-1}m$ at $T = 2$ K for the (10, 6) heterostructure (Fig. S20b), which is an order of magnitude smaller, presumably due to the much weaker and more inhomogeneous superconductivity in thinner samples.

5. Role of self-heating-induced thermal gradient in $R^{2\omega}$

In our second-harmonic measurements, an excitation current I_0 ~500 μA is used, corresponding to a current density i of ~ 1 A/m. The associated Joule heating, which also scales with I_0^2 and may contribute to the measured $R^{2\omega}$ signal. In this section, we evaluate the self-heating contribution to the observed nonreciprocal transport in1T-$CrTe_2$/FeTe heterostructures.

With an a.c. current i applied to the sample, a thermal gradient ΔT across the sample induced by self-heating {i.e., $\Delta T \propto i^2 R^\omega \propto p \cdot R^\omega \cdot [1+\sin(2\omega t - \pi/2)]$, where p is a constant related to the heating power} can change the sample resistance $R^\omega(T)$, which may in turn influence the measured magneto-chiral anisotropy coefficient γ. According to Eq. 1 in the main text, when the magneto-chiral anisotropy effect is included, the longitudinal voltage across the sample can be written as

$$V_{xx} = R^\omega I + \gamma R^\omega \left[(\mu_0 \overrightarrow{H_\parallel} \times \hat{z}) \cdot \vec{I} \right] I. \quad \text{(S4)}$$

When $\mu_0 H_\parallel$ is confined within the sample plane (i.e., perpendicular to $\hat{z}$), Eq. R4 simplifies to

$$V_{xx} = R^\omega I + \gamma R^\omega \mu_0 H_\parallel \cos\phi \, I^2. \quad \text{(S5)}$$

Taking into account the ΔT-induced resistance fluctuation $R^\omega(T+\Delta T) = R^\omega + (\partial R^\omega/\partial T) \cdot \Delta T$ yields

$$\begin{aligned} V_{xx} &= [R^\omega + (\partial R^\omega/\partial T)\Delta T]I + \gamma[R^\omega + (\partial R^\omega/\partial T)\Delta T]\mu_0 H_\parallel \cos\phi \, I^2 \\ &= \{R^\omega + (\partial R^\omega/\partial T) \cdot p \cdot R^\omega \cdot [1 + sin(2\omega t - \pi/2)]\}\sqrt{2} I_0 \sin(\omega t) \\ &+ \gamma\{R^\omega + (\partial R^\omega/\partial T) \cdot p \cdot R^\omega \\ &\cdot [1 + sin(2\omega t - \pi/2)]\}\mu_0 H_\parallel \cos\phi \, {I_0}^2 [1 + sin(2\omega t - \pi/2)] \\ &= \gamma R^\omega [1 + 3p(\partial R^\omega/\partial T)/2]\mu_0 H_\parallel \cos\phi \, {I_0}^2 \\ &+ \sqrt{2} R^\omega [1 + 3p(\partial R^\omega/\partial T)/2] I_0 \sin(\omega t) \\ &+ \gamma R^\omega [1 + 2p(\partial R^\omega/\partial T)]\mu_0 H_\parallel \cos\phi \, {I_0}^2 \sin(2\omega t - \pi/2) \\ &+ (\sqrt{2}/2) R^\omega (\partial R^\omega/\partial T) p I_0 \sin(3\omega t + \pi) \\ &+ (1/2)\gamma R^\omega (\partial R^\omega/\partial T) p \mu_0 H_\parallel \cos\phi \, {I_0}^2 \sin(4\omega t + \pi/2). \end{aligned} \quad \text{(S6)}$$

As a result, the first-harmonic response becomes $R^{\omega'} = \frac{\sqrt{2} R^\omega [1+3p(\partial R^\omega/\partial T)/2] I_0}{\sqrt{2} I_0} = R^\omega [1 + 3p(\partial R^\omega/\partial T)/2]$, and the second-harmonic response becomes $R^{2\omega'} =$

$\frac{\gamma R^{\omega}[1+2p(\partial R^{\omega}/\partial T)]\mu_0 H_{\parallel}\cos\phi I_0{}^2}{\sqrt{2}I_0} = \frac{\gamma R^{\omega}[1+2p(\partial R^{\omega}/\partial T)]\mu_0 H_{\parallel}\cos\phi I_0}{\sqrt{2}}$. Therefore, the ratio between $R^{2\omega\prime}$ and $R^{\omega\prime}$ is

$$R^{2\omega\prime}\Big/R^{\omega\prime} = \gamma\cdot\frac{\mu_0 H_{\parallel}\cos\phi\, I_0}{\sqrt{2}}\cdot\frac{1+2p(\partial R^{\omega}/\partial T)}{1+3p(\partial R^{\omega}/\partial T)/2}, \tag{S7}$$

and the magneto-chiral anisotropy coefficient γ can be expressed as

$$\begin{aligned}\gamma &= \frac{1+3p(\partial R^{\omega}/\partial T)/2}{1+2p(\partial R^{\omega}/\partial T)}\frac{\sqrt{2}}{\cos\phi\, I_0}\frac{R^{2\omega}/R^{\omega}}{\mu_0 H_{\parallel}} \\ &= \left\{1-\frac{1}{2\left[2+\frac{1}{p(\partial R^{\omega}/\partial T)}\right]}\right\}\frac{\sqrt{2}}{\cos\phi\, I_0}\frac{R^{2\omega}/R^{\omega}}{\mu_0 H_{\parallel}}.\end{aligned} \tag{S8}$$

Compared to the ideal case without self-heating in Section Ⅲ.2, the extracted γ-value is reduced by a factor $\alpha = 1-\frac{1}{2\left[2+\frac{1}{p(\partial R^{\omega}/\partial T)}\right]}$, which depends explicitly on $\partial R^{\omega}/\partial T$.

To evaluate the self-heating contribution to the observed nonreciprocal transport in the (10, 20) heterostructure, we plot T dependence of $\partial R^{\omega}/\partial T$ and α (Fig. S12a,b). We find that at low T, (i.e., $T \to T_{BKT}$), $\partial R^{\omega}/\partial T$ is negligibly small and α is ~1, suggesting that it remains appropriate to extract the γ values from the slopes of $R^{2\omega}/R^{\omega}$-$\mu_0 H_{\parallel}$ curves. As T increases, $\partial R^{\omega}/\partial T$ gradually increases and reaches its maximum at $T \sim T_{c,half}$, leading to a reduction of the extracted γ values. This effect may account for the deviation observed in the fitted curve in Fig. S12a.

As the largest γ values are observed when T approaches the BKT transition temperature T_{BKT}, where the residual resistances of the samples are extremely small. Specifically, R_{xx} = 0.063 Ω for the (10, 20) heterostructure at T = 11.15 K (Fig. 4c); R_{xx} = 0.056 Ω for the (5, 40) heterostructure at T = 11.2 K (Fig. S13a); and R_{xx} = 0.045 Ω for the (3, 40) heterostructure at T = 10.9 K (Fig. S13b). Under these conditions, the Joule heating power is estimated to be on the order of ~10 nW,

which is negligibly small. Therefore, the magneto-chiral anisotropy-induced contribution dominates the measured $R^{2\omega}$ signal in this temperature regime.

6. Absence of the d.c. diode effect in 1T-$CrTe_2$/FeTe

Nonreciprocal transport in superconductors can be probed by measuring either the superconducting critical current I_c in the deep superconducting state (i.e., detecting the asymmetry in I_c for opposite current directions) or the second harmonic resistance $R^{2\omega}$ near the superconducting transition temperature (i.e., the method adopted in this work). While related, these two methods probe different aspects of nonreciprocal transport: $R^{2\omega}$ measurements provide a more sensitive and quantitative probe of nonreciprocal transport near superconducting T_c, while I_c measurements reveal the d.c. superconducting diode effect in the deep superconducting phase (i.e., $T \ll T_c$). Both methods have been widely adopted in previous studies[24-29].

In addition to the magneto-chiral effect discussed in main text, the nonreciprocal transport behavior in superconducting films has also been attributed to the existence of the Meissner screening current j_s, which arises to screen the external magnetic field and/or the magnetization of the adjacent layer (i.e., via vortex nucleation)[30,31]. However, the nonreciprocal transport observed our 1T-$CrTe_2$/FeTe heterostructures is unlikely to be associated to the vortex nucleation for following reasons:

First, in the vortex nucleation scenario, j_s depends on the strength of the applied magnetic field and/or the magnetization of the adjacent layer, but it is independent of the applied current I. However, in our experiments, the nonreciprocal transport is probed by the $R^{2\omega}$ measurements. Unlike in the deep superconducting state (i.e., $T \ll T_c$), the Meissner response during the superconducting transition is significantly weaker (Fig. 3b). Second, within the range of magnetic fields used in our experiments, we did not observe any vortex-like features in our MFM images

even in the superconducting state (i.e., $T = 8$ K). Third, the observed quadratic dependence of $V^{2\omega}$ on I cannot be well explained by the physical picture of vortex nucleation. Therefore, we tend to believe that the magneto-chiral anisotropy is the dominant mechanism for the nonreciprocal transport in our 1T-$CrTe_2$/FeTe heterostructures.

To further investigate the nonreciprocal transport behavior observed in our 1T-$CrTe_2$/FeTe heterostructures, we measure the I-V curves of the (10, 20) heterostructure under different $\mu_0 H_{\parallel}$ and $\mu_0 H_{\perp}$ (Fig. S21). No difference has been observed in the I-V curves obtained under opposite directions of either $\mu_0 H_{\parallel}$ (Fig. S21a) or $\mu_0 H_{\perp}$ (Fig. S21b). The critical current I_c remains unchanged upon reversing the direction of either $\mu_0 H_{\parallel}$ or $\mu_0 H_{\perp}$. We believe that the absence of the d.c. superconducting diode effect in the deep superconducting state is likely due to the following three aspects:

(1) The effective area of our 1T-$CrTe_2$/FeTe Hall bar devices is ~500 μm × 1000 μm, which is much larger than the size of the Hall bar devices of less than ~5 μm × 10 μm used in prior studies[27-29]. The larger Hall bar geometry results in a higher critical current I_c. In our 1T-$CrTe_2$/FeTe Hall bar devices, I_c exceeds ~18 mA at $T = 8$ K without a magnetic field. Such a large I_c makes it challenging to resolve the subtle difference between I_{c+} and I_{c-}.

(2) As the sample size grows, its inhomogeneity becomes weaker due to the averaging of vortex motion, resulting in a more homogeneous vortex dynamics and the reduction of directional asymmetry. Although the inhomogeneity of the Meissner response has been observed in our samples over a length scale of ~10 μm, it becomes indiscernible after averaging over a length scale of 500~1000 mm. As the d.c. diode effect may result from the vortex nucleation, which requires the inhomogeneity of the sample, especially near its edges[30,31], the reduced inhomogeneity in our large-scale samples may lead to a weaker or even disappearing d.c. diode effect.

(3) As shown in Fig. S17, the upper critical magnetic field of the emergent superconductivity in our 1T-$CrTe_2$/FeTe heterostructures is more than 20 T at T = 8 K for both in-plane and out-of-plane magnetic fields. Therefore, the application of up to ±9 T has little impact on the emergent superconductivity. This may also obscure the manifestation of the d.c. superconducting diode effect in our 1T-$CrTe_2$/FeTe heterostructures.

7. Discussion on the superconducting mechanism of FeTe-based heterostructures

As noted in main text, superconductivity has been observed in a series of TI/FeTe heterostructures[7,19-22], though its origin remains unconclusive. Given the fact that FeTe is an antiferromagnetic iron chalcogenide which is non-superconducting without element doping (including O, S, or Se)[32-34] or tensile stress[35]. In our MBE chambers, because no other chalcogenide elements have been used and no discernable element diffusion has been resolved by EDS (Figs. 1e and S3), we can safely rule out element doping for the emergent superconductivity. To investigate the interfacial strain effect in reported superconducting FeTe-based heterostructures[7,19-22], we summarize the lattice constants of FeTe and it partner materials in Table S1. We find these partner layers are van der Waals materials and share a trigonal lattice structure. Given the structural mismatch between the trigonal partner layers and the tetragonal FeTe, it is not clear whether the interfacial strain between FeTe and its partner layer is tensile or compressive. More experimental works are needed to determine whether tensile or compressive stress is the primary origin of the emergent superconductivity in FeTe-based heterostructures.

As for the charge transfer between the FeTe layer and its partner layer, such interfacial charge transfer typically arises from differences in work function between two dissimilar materials. We summarize the work functions of the partner layers in Table S2. Although the work functions vary significantly among these partner layers, interface-induced superconductivity consistently

emerges in all FeTe-based heterostructures. This suggests that, while charge transfer may modulate the interfacial electronic band structure, it is unlikely to be the primary origin of the emergent superconductivity in FeTe-based heterostructures. Moreover, charge transfer alone cannot suppress the antiferromagnetic order in the bottom FeTe layer.

We note that a common feature among all superconducting FeTe-based heterostructures is the presence of a Te-based compound as the top partner layer, suggesting a potential correlation between the Te element and the emergence of superconductivity in FeTe-based heterostructures. We hypothesize that Te in the partner layer may suppress the antiferromagnetic order in FeTe during the MBE growth. This process makes the bottom FeTe layer itself become a superconductor. More experimental studies are needed to test this hypothesis.

8. Discussion on the a.c. frequency dependence of nonreciprocal transport

In most of our second-harmonic transport measurements, we use an a.c. current with the frequency of ~6.447 Hz (Methods). We choose this low frequency to minimize signal noise and reduce parasitic contributions in our a.c. measurement setup. In particular, inductive components in the circuit, which arise from long wires, sample contacts, and surrounding electronics, generate spurious voltage signals that scale with the a.c. frequency. By employing a low frequency of the excitation current, we effectively suppress these inductive artifacts, thereby improving the signal-to-noise ratio and isolating the true second-harmonic response associated with nonreciprocal charge transport in our 1T-$CrTe_2$/FeTe heterostructures.

Our nonreciprocal charge transport measurements show that the second-harmonic signal is frequency-independent when the frequency is less than 101.447 Hz (Fig. S10c). This frequency independence indicates that the observed nonreciprocal transport is an intrinsic property of our 1T-$CrTe_2$/FeTe heterostructures, rather than an artifact arising from inductive effects in the a.c.

measurement. We note that low a.c. excitation frequencies of less than 15 Hz have been commonly used in prior nonreciprocal transport studies[26,36,37].

References

1 Zhang, X., Lu, Q., Liu, W., Niu, W., Sun, J., Cook, J., Vaninger, M., Miceli, P. F., Singh, D. J., Lian, S. W., Chang, T. R., He, X., Du, J., He, L., Zhang, R., Bian, G. & Xu, Y. Room-temperature intrinsic ferromagnetism in epitaxial $CrTe_2$ ultrathin films. *Nat. Commun.* **12**, 2492 (2021).

2 Ou, Y., Yanez, W., Xiao, R., Stanley, M., Ghosh, S., Zheng, B., Jiang, W., Huang, Y. S., Pillsbury, T., Richardella, A., Liu, C., Low, T., Crespi, V. H., Mkhoyan, K. A. & Samarth, N. $ZrTe_2/CrTe_2$: an epitaxial van der Waals platform for spintronics. *Nat. Commun.* **13**, 2972 (2022).

3 Cheng, L., Liu, H. J., Zhang, J., Wei, J., Liang, J. H., Shi, J. & Tang, X. F. Effects of van der Waals interactions and quasiparticle corrections on the electronic and transport properties of Bi_2Te_3. *Phys. Rev. B* **90**, 085118 (2014).

4 Chen, X., Zhou, H. D., Kiswandhi, A., Miotkowski, I., Chen, Y. P., Sharma, P. A., Lima Sharma, A. L., Hekmaty, M. A., Smirnov, D. & Jiang, Z. Thermal expansion coefficients of Bi_2Se_3 and Sb_2Te_3 crystals from 10 K to 270 K. *Appl. Phys. Lett.* **99**, 261912 (2011).

5 Gnezdilov, V., Pashkevich, Y., Lemmens, P., Gusev, A., Lamonova, K., Shevtsova, T., Vitebskiy, I., Afanasiev, O., Gnatchenko, S., Tsurkan, V., Deisenhofer, J. & Loidl, A. Anomalous optical phonons in FeTe chalcogenides: Spin state, magnetic order, and lattice anharmonicity. *Phys. Rev. B* **83**, 245127 (2011).

6 Trang, C. X., Li, Q., Yin, Y., Hwang, J., Akhgar, G., Di Bernardo, I., Grubisic-Cabo, A., Tadich, A., Fuhrer, M. S., Mo, S. K., Medhekar, N. V. & Edmonds, M. T. Crossover from 2D Ferromagnetic Insulator to Wide Band Gap Quantum Anomalous Hall Insulator in Ultrathin $MnBi_2Te_4$. *ACS Nano* **15**, 13444-13452 (2021).

7 Yi, H., Hu, L. H., Zhao, Y. F., Zhou, L. J., Yan, Z. J., Zhang, R., Yuan, W., Wang, Z., Wang, K., Hickey, D. R., Richardella, A. R., Singleton, J., Winter, L. E., Wu, X., Chan, M. H. W., Samarth, N., Liu, C. X. & Chang, C. Z. Dirac-fermion-assisted interfacial superconductivity in epitaxial topological-insulator/iron-chalcogenide heterostructures. *Nat. Commun.* **14**, 7119 (2023).

8 Choudhary, K., Garrity, K. F., Reid, A. C. E., DeCost, B., Biacchi, A. J., Hight Walker, A. R., Trautt, Z., Hattrick-Simpers, J., Kusne, A. G., Centrone, A., Davydov, A., Jiang, J., Pachter, R., Cheon, G., Reed, E., Agrawal, A., Qian, X., Sharma, V., Zhuang, H., Kalinin,

S. V., Sumpter, B. G., Pilania, G., Acar, P., Mandal, S., Haule, K., Vanderbilt, D., Rabe, K. & Tavazza, F. The joint automated repository for various integrated simulations (JARVIS) for data-driven materials design. *Npj Comput. Mater.* **6**, 173 (2020).

9 Akhgar, G., Li, Q., Di Bernardo, I., Trang, C. X., Liu, C., Zavabeti, A., Karel, J., Tadich, A., Fuhrer, M. S. & Edmonds, M. T. Formation of a Stable Surface Oxide in $MnBi_2Te_4$ Thin Films. *ACS Appl. Mater. Interfaces* **14**, 6102-6108 (2022).

10 Thiel, S., Hammerl, G., Schmehl, A., Schneider, C. W. & Mannhart, J. Tunable quasi-two-dimensional electron gases in oxide heterostructures. *Science* **313**, 1942-1945 (2006).

11 Gozar, A., Logvenov, G., Kourkoutis, L. F., Bollinger, A. T., Giannuzzi, L. A., Muller, D. A. & Bozovic, I. High-temperature interface superconductivity between metallic and insulating copper oxides. *Nature* **455**, 782-785 (2008).

12 Reyren, N., Thiel, S., Caviglia, A. D., Kourkoutis, L. F., Hammerl, G., Richter, C., Schneider, C. W., Kopp, T., Ruetschi, A. S., Jaccard, D., Gabay, M., Muller, D. A., Triscone, J. M. & Mannhart, J. Superconducting interfaces between insulating oxides. *Science* **317**, 1196-1199 (2007).

13 Liu, C., Yan, X., Jin, D., Ma, Y., Hsiao, H. W., Lin, Y., Bretz-Sullivan, T. M., Zhou, X., Pearson, J., Fisher, B., Jiang, J. S., Han, W., Zuo, J. M., Wen, J., Fong, D. D., Sun, J., Zhou, H. & Bhattacharya, A. Two-dimensional superconductivity and anisotropic transport at $KTaO_3$ (111) interfaces. *Science* **371**, 716-721 (2021).

14 Harper, F. E. & Tinkham, M. The Mixed State in Superconducting Thin Films. *Phys. Rev.* **172**, 441-450 (1968).

15 Uchihashi, T. Two-dimensional superconductors with atomic-scale thickness. *Supercond. Sci. Technol.* **30** (2017).

16 Fink, H. J. Vortex Nucleation in a Superconducting Slab near a Second-Order Phase Transition and Excited States of the Sheath near H_{c3}. *Phys. Rev.* **177**, 732-737 (1969).

17 Lei, H., Hu, R., Choi, E. S., Warren, J. B. & Petrovic, C. Effects of excess Fe on upper critical field and magnetotransport in $Fe_{1+y}(Te_{1-x}S_x)_z$. *Phys. Rev. B* **81**, 184522 (2010).

18 Fuchs, G., Drechsler, S. L., Kozlova, N., Behr, G., Kohler, A., Werner, J., Nenkov, K., Klingeler, R., Hamann-Borrero, J., Hess, C., Kondrat, A., Grobosch, M., Narduzzo, A., Knupfer, M., Freudenberger, J., Buchner, B. & Schultz, L. High-field pauli-limiting behavior and strongly enhanced upper critical magnetic fields near the transition

temperature of an arsenic-deficient $LaO_{0.9}F_{0.1}FeAs_{1-\delta}$ superconductor. *Phys. Rev. Lett.* **101**, 237003 (2008).

19 He, Q. L., Liu, H., He, M., Lai, Y. H., He, H., Wang, G., Law, K. T., Lortz, R., Wang, J. & Sou, I. K. Two-dimensional superconductivity at the interface of a Bi_2Te_3/FeTe heterostructure. *Nat. Commun.* **5**, 4247 (2014).

20 Liang, J., Zhang, Y. J., Yao, X., Li, H., Li, Z. X., Wang, J., Chen, Y. & Sou, I. K. Studies on the origin of the interfacial superconductivity of $Sb_2Te_3/Fe_{1+y}Te$ heterostructures. *PNAS* **117**, 221-227 (2020).

21 Yi, H., Zhao, Y. F., Chan, Y. T., Cai, J., Mei, R., Wu, X., Yan, Z. J., Zhou, L. J., Zhang, R., Wang, Z., Paolini, S., Xiao, R., Wang, K., Richardella, A. R., Singleton, J., Winter, L. E., Prokscha, T., Salman, Z., Suter, A., Balakrishnan, P. P., Grutter, A. J., Chan, M. H. W., Samarth, N., Xu, X., Wu, W., Liu, C. X. & Chang, C. Z. Interface-induced superconductivity in magnetic topological insulators. *Science* **383**, 634-639 (2024).

22 Yuan, W., Yan, Z. J., Yi, H., Wang, Z., Paolini, S., Zhao, Y. F., Zhou, L., Wang, A. G., Wang, K., Prokscha, T., Salman, Z., Suter, A., Balakrishnan, P. P., Grutter, A. J., Winter, L. E., Singleton, J., Chan, M. H. W. & Chang, C. Z. Coexistence of Superconductivity and Antiferromagnetism in Topological Magnet $MnBi_2Te_4$ Films. *Nano Lett.* **24**, 7962-7971 (2024).

23 Sun, Y. Z., Yan, P. F., Ning, J. A., Zhang, X. Q., Zhao, Y. F., Gao, Q. W., Kanagaraj, M., Zhang, K. P., Li, J. J., Lu, X. Y., Yan, Y., Li, Y., Xu, Y. B. & He, L. Ferromagnetism in two-dimensional $CrTe_2$ epitaxial films down to a few atomic layers. *AIP Adv.* **11**, 035138 (2021).

24 Wakatsuki, R., Saito, Y., Hoshino, S., Itahashi, Y. M., Ideue, T., Ezawa, M., Iwasa, Y. & Nagaosa, N. Nonreciprocal charge transport in noncentrosymmetric superconductors. *Sci. Adv.* **3**, e1602390 (2017).

25 Hoshino, S., Wakatsuki, R., Hamamoto, K. & Nagaosa, N. Nonreciprocal charge transport in two-dimensional noncentrosymmetric superconductors. *Phys. Rev. B* **98**, 054510 (2018).

26 Yasuda, K., Yasuda, H., Liang, T., Yoshimi, R., Tsukazaki, A., Takahashi, K. S., Nagaosa, N., Kawasaki, M. & Tokura, Y. Nonreciprocal charge transport at topological insulator/superconductor interface. *Nat. Commun.* **10**, 2734 (2019).

27 Bauriedl, L., Bauml, C., Fuchs, L., Baumgartner, C., Paulik, N., Bauer, J. M., Lin, K. Q., Lupton, J. M., Taniguchi, T., Watanabe, K., Strunk, C. & Paradiso, N. Supercurrent diode effect and magnetochiral anisotropy in few-layer $NbSe_2$. *Nat. Commun.* **13**, 4266 (2022).

28 Jeon, K. R., Kim, J. K., Yoon, J., Jeon, J. C., Han, H., Cottet, A., Kontos, T. & Parkin, S. S. P. Zero-field polarity-reversible Josephson supercurrent diodes enabled by a proximity-magnetized Pt barrier. *Nat. Mater.* **21**, 1008-1013 (2022).

29 Kim, J. K., Jeon, K. R., Sivakumar, P. K., Jeon, J., Koerner, C., Woltersdorf, G. & Parkin, S. S. P. Intrinsic supercurrent non-reciprocity coupled to the crystal structure of a van der Waals Josephson barrier. *Nat. Commun.* **15**, 1120 (2024).

30 Hou, Y., Nichele, F., Chi, H., Lodesani, A., Wu, Y., Ritter, M. F., Haxell, D. Z., Davydova, M., Ilic, S., Glezakou-Elbert, O., Varambally, A., Bergeret, F. S., Kamra, A., Fu, L., Lee, P. A. & Moodera, J. S. Ubiquitous Superconducting Diode Effect in Superconductor Thin Films. *Phys. Rev. Lett.* **131**, 027001 (2023).

31 Gutfreund, A., Matsuki, H., Plastovets, V., Noah, A., Gorzawski, L., Fridman, N., Yang, G., Buzdin, A., Millo, O., Robinson, J. W. A. & Anahory, Y. Direct observation of a superconducting vortex diode. *Nat. Commun.* **14**, 1630 (2023).

32 Mizuguchi, Y., Tomioka, F., Tsuda, S., Yamaguchi, T. & Takano, Y. Superconductivity in S-substituted FeTe. *Appl. Phys. Lett.* **94**, 012503 (2009).

33 Nie, Y. F., Telesca, D., Budnick, J. I., Sinkovic, B., Ramprasad, R. & Wells, B. O. Superconductivity and properties of $FeTeO_x$ films. *J. Phys. Chem. Solids* **72**, 426-429 (2011).

34 Li, F., Ding, H., Tang, C., Peng, J., Zhang, Q., Zhang, W., Zhou, G., Zhang, D., Song, C.-L., He, K., Ji, S., Chen, X., Gu, L., Wang, L., Ma, X.-C. & Xue, Q.-K. Interface-enhanced high-temperature superconductivity in single-unit-cell $FeTe_{1-x}Se_x$ films on $SrTiO_3$. *Phys. Rev. B* **91**, 220503(R) (2015).

35 Han, Y., Li, W. Y., Cao, L. X., Wang, X. Y., Xu, B., Zhao, B. R., Guo, Y. Q. & Yang, J. L. Superconductivity in iron telluride thin films under tensile stress. *Phys. Rev. Lett.* **104**, 017003 (2010).

36 Choe, D., Jin, M. J., Kim, S. I., Choi, H. J., Jo, J., Oh, I., Park, J., Jin, H., Koo, H. C., Min, B. C., Hong, S., Lee, H. W., Baek, S. H. & Yoo, J. W. Gate-tunable giant nonreciprocal charge transport in noncentrosymmetric oxide interfaces. *Nat. Commun.* **10**, 4510 (2019).

37 Itahashi, Y. M., Ideue, T., Saito, Y., Shimizu, S., Ouchi, T., Nojima, T. & Iwasa, Y. Nonreciprocal transport in gate-induced polar superconductor $SrTiO_3$. *Sci. Adv.* **6**, eaay9120 (2020).
